# Breeding Climate-Resilient Soybeans for 2050 and Beyond: Leveraging Novel Technologies to Mitigate Yield Stagnation and Climate Change Impacts

**DOI:** 10.3390/plants15081201

**Published:** 2026-04-14

**Authors:** Muhammad Amjad Nawaz, Gyuhwa Chung, Igor Eduardovich Pamirsky, Kirill Sergeevich Golokhvast

**Affiliations:** 1 Higher Engineering School of Agrobiotechnology, National Research Tomsk State University, Lenin Ave, 36, 634050 Tomsk, Russia; 2 Laboratory for Research and Application of Supercritical Fluid Technologies in Agro-Food Biotechnology, National Research Tomsk State University, Lenin Ave, 36, 634050 Tomsk, Russia; 3 Korea Soybean Research Institute Inc., Jinju 52670, Republic of Korea; 4 Siberian Federal Scientific Centre of Agrobiotechnology, RAS, 7 Centralnaya Street, Presidium, 633501 Krasnoobsk, Russia

**Keywords:** climate change, future plant breeder, molecular breeding, plant architecture, soybean breeding, 2050, stress physiology, traits of interest

## Abstract

Soybean is a vital crop supporting global food, feed, and biofuel production. Soybean yields have surged, with record yields reaching 14,678 kg/ha^−1^, though average farm yields remain stagnant at 2770–2790 kg ha^−1^. The persistent yield gaps leave 44% of potential production unrealized due to climate change, threatening food security. To meet future caloric demands, which are projected to rise by 46.8% by 2050, soybean breeding must prioritize climate-resilient, high-yielding varieties with minimal ecological footprints. In this comprehensive and in-depth review, we synthesized existing literature and Google Patents and reviewed the multifaceted impacts of climate-change driven eCO_2_ and stresses (heat, drought, flooding, salinity, and pathogens), revealing non-linear interactions where eCO_2_ may not compensate yield losses under combined stresses. We then highlight key strategies for soybean breeding under climate-change scenario. To this regard, we provide a detailed trait-by-trait breeding roadmap covering seed number, seed size, seed weight, protein-oil balance and their metabolic trade-offs, above and below ground plant architecture, nitrogen fixation and nodulation dynamics, root system architecture, water use efficiency, canopy architecture, flowering time regulation, early maturity etc., in light of specific genes and validated strategies. We explicitly discuss the novel strategies including deeper understanding of traits, abiotic stress physiology, changing pathogen dynamics, phenomics, (multi-)omics, machine learning, and modern biotechnological techniques for developing future soybean varieties. We provide a future roadmap prioritizing specific actions, including engineering climate-resilient ideotypes through gene stacking, optimizing nitrogen fixation and nutrition under stresses leveraging omics data, pan-genome, wild soybean, speeding breeding hubs, and participatory farmer-network validation, while redefining the future soybean breeder would be a hybrid orchestrator of data and dirt. This review establishes a foundational framework for translating climate-adaptive morphological, biochemical, physiological, omics, agronomic, phenomics, and biotechnological insights into actionable breeding strategies, thereby guiding policy-driven investment in soybean improvement programs targeting 2050 and beyond.

## 1. Introduction

**Figure 1:**
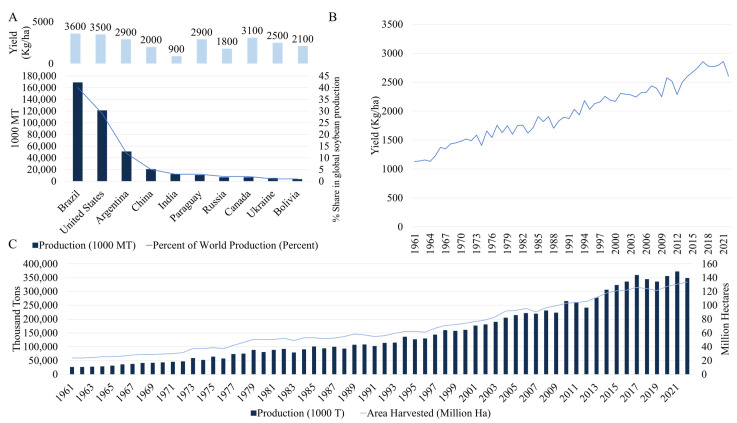
Soybean production statistics. (**A**) Production and yield in top-10 soybean producing countries. (**B**) Global soybean yield (kg ha^−1^) (1961–2021). (**C**) Global area harvested and production of soybean (1961–2022). Data source: https://ipad.fas.usda.gov/; (accessed on 2 July 2024) and [5,6].

Soybean [*Glycine max* (L.) Merr.] is an annual legume species which was domesticated from *Glycine soja* Sieb. & Zucc. Soybeans were originally cultivated in East Asian countries i.e., China (as early as 5000 years ago), Taiwan, Korea, and Japan. It remained a predominantly Asian crop until 1740s, when it was sent to Europe possibly by missionaries and in 1760s to America (Georgia) [1]. As of 2025, soybean is cultivated in more than 100 countries with Brazil being the top producer with 169,000 thousand metric tons in 2024–25 (40% of global production), followed by United States of America (USA), Argentina, and China (Figure 1; https://ipad.fas.usda.gov/; accessed on 2 July 2024). Global soybean production in 2024–25 was 420,870 thousand metric tons, up from 396,930 thousand metric tons in 2023–24 [2]. Soybeans are used in processed form (~87%, animal feed, biofuels, vegetable oil, etc.), direct animal feed (~8.4%), or direct human food (~3.8%, tofu, soy milk, tempeh, etc.). Soybeans are consumed because of their nutritional value as they contain protein (35%), carbohydrates (25–30%), and other health improving compounds i.e., polyunsaturated fatty acids (FAs), antioxidants, minerals, vitamins, and fiber [3]. Due to presence of a range of bioactive compounds such as biopeptides, isoflavones, lecithin, and saponins, soybeans are implicated in protective effects on health [4].

By 2050, soybean breeders must overcome major challenges associated with climate resilience, evolving pests/diseases, yield stagnation, and nutritional demands. Soybean breeders have been working on adaptation since ancient China’s selective cultivation and 19th century breakthroughs in germplasm exchange, biochemistry, and agronomy. Early efforts, from Qing Dynasty’s varietal documentation to transcontinental introductions and nitrogen fixation research, laid the foundation for modern breeding [1,7,8].

Today, integrating cutting-edge biotech with historical knowledge is critical to future-proof soybean production amid climate change and global food needs. Historically, with the invent of genetic engineering and genomic technologies, the focus has shifted towards stress tolerance and value-addition. A google patent search (1900–2025) against keywords (soybean, *Glycine max*, variety, development) indicated 2240 results (https://patents.google.com/?q=(soybean%2c+variety%2c+development%2c+Glycine+max)&before=priority:20250526&after=priority:19000101&type=PATENT&num=100&clustered=true; accessed on 26 May 2025). The prevalent technologies in the varietal development include genetic engineering, marker assisted selection (MAS), conventional breeding, gene editing, etc. Whereas the breeding methods include cross-breeding, backcrossing, genomic selection (GS), and phenotyping. The most focused traits include herbicide tolerance, insect resistance, disease resistance, followed by agronomic traits (pod-shattering, drought/salt tolerance, high-oleic/low-linolenic oil, etc.), microbial and non-GMO solutions (seed coating with beneficial microbes and nitrogen-fixing bacteria), and precision agriculture and AI (high-throughput phenotyping (e.g., hyperspectral imaging) and automated seed sampling). USA, China, EU, and Brazil are the patent filing hotspots with the private industry leading the innovation.

Nevertheless, the adoption of modern breeding technologies has driven a substantial increase in global soybean yield from 1128.7 to 2870–2900 Kg ha^−1^ in 2024/25 [9]. While this trend is global, leading producers such as USA, EU, Brazil (particularly USA, yields have increased by approximately 370% (from 740 kg ha^−1^ to 3470 kg ha^−1^)) have been at the forefront of developing new varieties focused on yield improvement, resistance to pests, diseases, and abiotic stresses, and improving seed composition for higher protein and oil contents. This has been achieved by the implementation of technological waves: initial progress was driven by classical breeding and genetics in early decades of the last century, followed by a widespread adoption of biotechnology-driven herbicide tolerance traits from 1996 onward, complemented by advances in agricultural mechanization and management. Most recently, the integration of genomics, phenomics, and advanced analytics has further accelerated genetic improvement [10].

In case of East Asian countries i.e., China, Japan, and South Korea, yields have risen significantly from 600, 600, and 1300 Kg ha^−1^ (in 1961) to 2001, 1770, and 2090 Kg ha^−1^ (in 2024/25), respectively. This has been facilitated by the use of traditional breeding methods as well as recent technologies such as MAS and GS for complex traits, the implementation of rapid-generation breeding in controlled CO_2_-supplemented growth chambers, and genome editing (e.g., Clustered regularly interspaced short palindromic repeats (CRISPR)/CRISPR-associated nuclease (CRISPR-Cas9) and TALENs) to optimize key genes for yield-related traits such as flowering time and plant architecture [11]. Reported yield gains in China are mainly due to improvements in seed-related traits and canopy architecture, which enhance overall biomass accumulation [12]. Quantitatively, studies from different countries have reported positive genetic gains of ∼6 to 45 Kg ha^−1^yr^−1^ [13]. However, a maximum genetic gain of 84.3 Kg ha^−1^yr^−1^ has been reported from specific regional studies in Southern Brazil with a particular focus on maturity groups and evaluation periods [13]. The table below summarizes some studies estimating genetic gains in major producer countries (Table 1); reflecting overall progress, which often include both GM and non-GM varieties. For example, USA, Brazil, Argentina, Canada, Paraguay, and Ukraine have reported adaption of GM soybean (or both GM and non-GM), contributing to yield gains through improved weed and pest management. Contrastingly, China, India, and Russia have reported predominant use of non-GM soybean. Direct comparison of genetic gains between GM and non-GM systems is difficult as most original studies report aggregate gains. It is important to note that the realized genetic gain is highly dependent on genomic prediction models, selection intensity, and breeding strategy, all of which can also influence the genetic gain and genetic erosion in soybean breeding [14].

## 2. Yield Stagnation: Pressure, Pattern, and Production Risks

Soybean production faces significant challenges in addressing global food security, with persistent yield gaps and climate change-driven stagnation. As an important crop that contributes 8% of global caloric intake, soybean’s production trends reveal patterns that seek researchers’ attentions. While, record yields have reached 14,678 ~kg/ha^−1^ in the contest plots mainly because of high input and zero stress [34], although, the reported theoretical potential is 7250–11,000 kg ha^−1^, which is based on biophysical models. Yet, the average farm yields remain stagnant at 2770–2790 kg ha^−1^ globally. Achieving theoretical yield potential in standard commercial field production remains highly challenging and rare. In practice, substantial yield gaps exist between what is physiologically possible and what is harvested on average farms. Regional analyses show 23% of global harvested area (19 million ha) has been experiencing yield stagnation, mainly in major producing regions including China (where stagnation affects significant portions of growing areas), India, the US (particularly fringe regions of the Corn Belt), Paraguay, Brazil, and Argentina [35]. This stagnation occurs amidst widening yield gaps across 37% of production zones since 1975, creating a paradox where improved agricultural technologies coexist with unrealized yield potential. Climate change exacerbates these challenges through multiple pathways: temperature extremes (yield losses of 45.5% above 28 °C [36]), cold stress (yield loss up to 24% [37]), and water-related stresses i.e., drought (yield loss of 28–74% [38]) and flooding (yield loss of 17–56% [39]). These abiotic stresses may also interact with biotic stresses, such that diseases become more prevalent under warmer, wetter conditions e.g., sudden death syndrome (SDS), while others e.g., charcoal rot, thrive in drought-stressed plants [40]. The CO_2_ fertilization effect (see Section 3 for details), while potentially offsetting some losses by enhancing photosynthesis, may reduce seed protein content and fails to compensate for extreme weather impacts. Multi-model projections still show 0.43–19.51% yield declines by 2050 under various climate scenarios (RCP2.6 to RCP8.5), with Brazil and the U.S. facing particularly severe losses up to 27.89% and 18.05%, respectively, in high-emission scenarios [41]. These production challenges emerge against a backdrop of soaring demand, with global caloric needs projected to grow 46.8% by 2050, including 41.2% increases in lower-middle income countries and 31.6% in low-income nations compared to 2020 levels [41,42]. These circumstances create an urgent need to address yield gaps that currently leave 44% of potential production unrealized in key regions like Southern Brazil [43].

Moreover, the geographic concentration of production in climate-vulnerable regions creates systemic risks for global food systems, with modeling showing how yield reduction transmits through international markets. Under RCP8.5 scenarios, climate impacts could reduce soybean supply by 14.2% in Bolivia and 2.27% in China (despite some local yield gains from warming at higher latitudes), leading to increased price (up to 91%) in import-dependent countries like India [41]. While trade liberalization may marginally boost flows (5–10%) through tariff elimination and improved infrastructure, it cannot fully compensate for production declines in major exporters like the U.S. and Brazil, which together dominate global supply. This vulnerable production scenario demands integrated solutions addressing both biological and socioeconomic constraints. Genetic innovation must simultaneously target multiple stress tolerances—including heat-resilient flowering stability (>25 °C), disease resistance (particularly against SDS and charcoal rot), and maintained nutritional quality under elevated CO_2_ (eCO_2_) [41,43]. Precision agronomic adaptations show particular promise, with optimized planting dates potentially recovering 18% of yields in tropical regions and improved water management mitigating drought impacts [44,45]. However, realizing these gains requires overcoming systemic barriers including variable input access, knowledge dissemination gaps, and economic constraints faced by smallholder producers who cultivate significant portions of global soybean area. With 79% of current production coming from yield-increasing areas that face growing climate risks [35], and with caloric demands rising fastest precisely in those nations who are least able to absorb price shocks (41.2% increase needed in lower-middle income countries) [41], the window for action is narrowing.

## 3. Impact of Climate Change on Soybean Yield and Productivity

Climate change is a major concern of 21st century which continues to reshape earth’s ecosystem. Climate change is a long-term shift in earth’s average weather patterns and temperature, majorly driven by anthropogenic activities (https://www.un.org/en/climatechange/what-is-climate-change; accessed on 28 November 2025). This results in the release of greenhouse gases e.g., CO_2_ and methane, which trap heat and cause the global warming, leading to extreme weather, rising sea level, floods, droughts, irregular precipitation patterns, heat waves, and melting ice [46]. It has comprehensive impacts on agriculture and its far-reaching effects are now clearly visible on crop production. One-third of the global soybean production failure in 2012 was attributed to climate change driven higher temperatures and stronger heat-moisture interactions [47]. Below we specifically review the effect of a major driver of climate change i.e., CO_2_ alone or combined with high temperature under different stress scenarios such as drought, flooding, salinity, and diseases, on soybean production.

### 3.1. Elevated CO_2_ Levels Affect Soybean Growth, Development, and Production in a Non-Linear Way

Carbon dioxide is a fundamental driver of climate change and its concentration continue to increase (~422.8 ppm in 2024 compared to ~294.22 ppm in 1900; https://www.climate.gov/; accessed on 29 May 2025). Soybean, as a C3 plant, generally exhibits increased growth and yield under eCO_2_ due to enhanced photosynthetic rates and improved water-use efficiency (WUE) from reduced stomatal conductance. Studies show that eCO_2_ promotes greater biomass accumulation, including higher total dry weight, increased number of pods and total leaf area, and elevated levels of sucrose and stachyose in seeds [48]. An increase in soybean yields (during 2002–2006) up to 5.84% has been reported on average as a result of CO_2_ increase from 1980 [49]. However, these benefits follow a nonlinear pattern—while photosynthesis and biomass initially rise with CO_2_ concentration, gains begin to diminish beyond ~900 ppm due to photosynthetic enzyme saturation and/or carbon sink limitations [50]. This plateau effect suggests that the widely assumed CO_2_ fertilization benefit is not universal; future CO_2_ increases may have progressively smaller impacts on soybean productivity, and breeding programs cannot rely on rising CO_2_ as primary driver of productivity gains. The positive effects of eCO_2_ are further influenced by competition, particularly with other C3 plants. For instance, while soybean biomass and yield increase in monoculture under eCO_2_ (ambient + 250 ppm), competition with weeds like *Chenopodium album* can significantly reduce these benefits [51]. These results carry important practical implications: weed management will become even more critical under future CO_2_ scenario, as the fertilization effect would also benefit competing weeds. Additionally, genotypic variation plays a role, with different soybean cultivars exhibiting distinct responses to CO_2_ enrichment [52]. Thus, the literature suggests that genetic selection for responsiveness to eCO_2_ could be viable breeding target, though this trait has received insufficient attention relative to stress tolerance.

**Figure 2:**
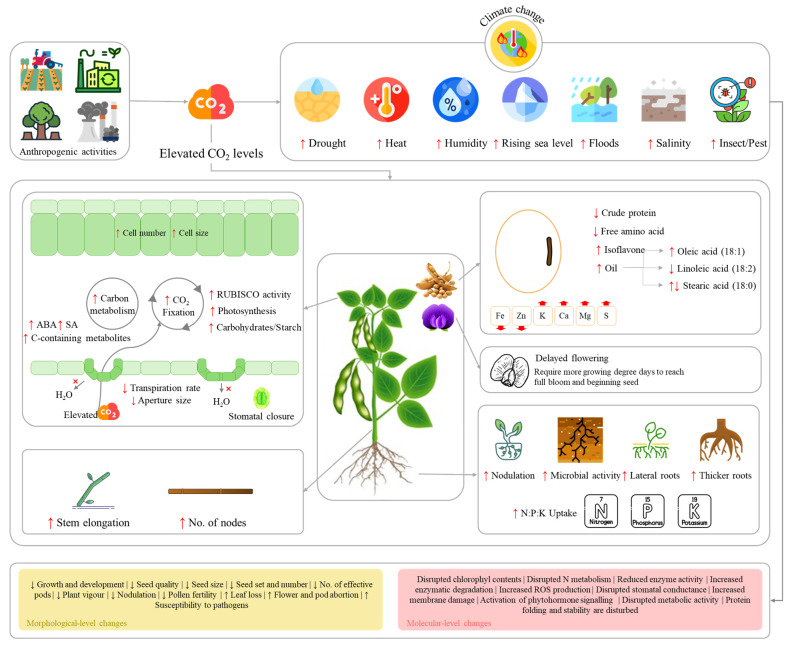
Impact of eCO_2_ and climate change drivers on soybean production. Anthropogenic activities (agriculture, deforestation, industrial exhaust, and burning fossil fuels) cause elevated CO_2_ levels in atmosphere, which in turn improve soybean growth and yield. eCO_2_ improves soybean growth by increasing photosynthesis [57], changes leaf structure [58], elongates stems and no. of nodes, increases nodulation [57], root microbial activity [59], increased lateral roots and thicker main roots [60]. Overall, increase in N, P, and K is observed. However, flowering is delayed in some genotypes and more growing degree days are required to reach the full bloom and seed setting [61]. eCO_2_ grown soybeans have reduced crude protein and free amino acids in seeds. The nutrient content exhibits complex changes and are genotype dependent [52,54,62]. However, oil and isoflavone contents are increased [52]. eCO_2_ causes climate change and increases the intensities of biotic and abiotic stresses. These stresses impact soybean morphology and physiology. ↑ and ↓ in figure panels indicate increase and decrease, respectively. Figure icons were downloaded from Flaticon (https://www.flaticon.com) and figure was prepared in Microsoft Power Point 2021 Professional Pro.

At the physiological level, eCO_2_ alters soybean development, delaying maturation while increasing plant height, node number, and leaf area [50,53]. Transcriptomic analyses have revealed that eCO_2_ upregulates genes associated with cell growth, carbohydrate metabolism, and respiration, supporting enhanced leaf expansion [53]. However, these morphological changes do not always translate to improved yield components, as excessive CO_2_ can reduce seed weight and alter harvest index [48]. This disconnect between vegetative vigor and reproductive output is a critical insight. It indicates that carbon allocation patterns under eCO_2_ may favor structural growth over grain fill, a physiological trade-off that breeders must actively manage. Seed quality is significantly affected by eCO_2_, with mature seeds (R8 stage) showing decreased crude protein and free amino acid concentrations but increased oil and isoflavone content [52]. Fatty acid composition shifts toward higher monounsaturated fats (e.g., oleic acid) in some cultivars, while mineral nutrition exhibits complex changes—declines in Fe and Zn but increases in K, Ca, Mg, and S at certain growth stages [52,54]. These alterations raise concerns about potential nutrient dilution in soybean-based foods under future CO_2_ levels. Modeling approaches, such as the modified GLYCIM simulator, demonstrate improved accuracy in predicting soybean gas exchange and biomass partitioning under eCO_2_, highlighting the importance of refining crop models for climate change scenarios [55]. However, field validations are needed to confirm these predictions, as chamber-based studies may not fully capture real-world interactions. This also identifies that continued reliance on chamber-based eCO_2_ studies is a methodological limitation that can be replaced by free-air CO_2_ enrichment facilities that better simulate field conditions. This concern is not unique to soybean as studies on wheat and soybean have shown that yield increase at eCO_2_ were larger in both crops when open top chambers were used than free-air CO_2_ enrichment [56]. Such discrepancies suggest that chamber-based studies may over-estimate the realized benefits of eCO_2_ under real-word filed conditions. Nevertheless, while eCO_2_ enhances soybean photosynthesis and growth, its benefits are constrained by diminishing returns at high concentrations, competition, and trade-offs in seed quality (Figure 2). Moreover, meta-analysis of eCO_2_ studies across 25 variables reported that the increased CO_2_ boosts photosynthesis but internal plant feedback and root limitations often prevent these gains from significantly increasing yield [57]. Therefore, the interplay between CO_2_ fertilization and these limiting factors will shape soybean productivity and nutritional value in a high-CO_2_ future.

### 3.2. eCO_2_ Combined with High Temperatures Significantly Amplifies the Negative Impacts of Climate Change on Soybean Crops

Elevated CO_2_ levels are causing an increase in average global temperatures; in 2025, the earth’s surface temperature was 2.11 °F above the 20th century average (https://www.ncei.noaa.gov/; accessed on 12 April 2026). Soybean growing areas have temperatures at or above physiological thresholds [63]. High temperatures can negatively impact soybean growth and development, especially during reproductive stages, leading to reduced yield, seed quality, and overall plant vigor. At morphological level pollen fertility is reduced, flowers and pods are aborted, or those which aren’t aborted have smaller seed size, lower seed weight, lower number of effective pods and seeds per plant, delayed plant development leading to reduced seed set, decreased nodulation, leaf loss, and overall seed quality. At molecular level, high temperature can disrupt chlorophyl content and photosynthesis, nitrogen metabolism, enzyme activity and degradation, reactive oxygen species (ROS) production, stomatal conductance and membrane damage, phytohormone synthesis and signaling, increased susceptibility to pathogens, metabolic pathways, protein folding and stability, etc. [64]. The data indicating that a 1 °C increase in temperature can lead to 17% decrease in soybean yield, provides a useful global benchmark. However, this figure likely underestimates yield losses in tropical regions, where the baseline temperatures already approach critical thresholds, while potentially overestimating losses in temperate regions where warming may relieve cold stress constraints. Thus, breeders must consider this spatial heterogeneity in temperature sensitivity, when setting regional breeding priorities. In soybean growing areas (e.g., of Canada), the mean temperature is expected to increase by 1.6, 2.8, and 4.1 °C during the growing season (May–September) in the near-term (2030s), mid-term (2050s), and distant future (2070s), respectively, under the SSP3-7.0 emission scenario [65]. Studies have shown that higher temperatures such as 26 °C, 29 °C, 32 °C, and 35 °C can reduce soybean yields by 8%, 14%, 51%, and 65%, respectively [66]. This response curve demonstrates a non-linear threshold behavior; that is, losses remain modest below 32 °C but more than double between 32 and 35 °C. This indicates an inflection point where reproductive processes i.e., pollen viability, fertilization, and seed set [67], begin to fail significantly. From a breeding perspective, raising the critical threshold from 32 to 35 °C would give better yield benefits; a more viable breeding objective rather than completely eliminating all heat sensitivity. Additionally, exceeding night temperatures than optimal thresholds are an added threat, impacting seed composition with carbohydrates being substantially affected than protein and oil [36]. Since most breeding programs screen for heat tolerance during daytime temperatures only, future breeding must incorporate night temperature screening protocols to achieve true-heat-resilience. Overall, global warming trend would negatively impact soybean yields, it would also favor soybean production expansion to northern and cooler regions (especially in Europe and Russia [68]). Warmer conditions could also reduce drought stress and improve yields in some western regions of Northeast China [69].

Most effects of individual stress are neutralized (buffering effect) when combined [70], though not universal, as sometimes, eCO_2_ may not compensate for the yield loss under high yield [48]. In other cases, an increase in photosynthetic rates (>900 ppm CO_2_) have been reported in response to higher temperature under eCO_2_ but not at ambient CO_2_ [71]. Such stark differences could be due to differences in plant nutrition, developmental stage under stress, vapor pressure, etc. [55]. For example, eCO_2_ together with higher temperature can increased P uptake because of increased root biomass and changes in root architecture [72]. On the contrary, reduced stomatal conductance is also reported, which improves WUE. Higher CO_2_ also increases biomass both in the total dry weight of plants and number of pods and leaves. Increased stachyose and sucrose levels have also been reported when soybean is grown under eCO_2_ levels (720 ppm) [48]. When combined with higher temperatures (optimum CO_2_ levels), 100-seed weight declines. Whereas, eCO_2_ levels (720 ppm) together with high temperatures (38/30 °C) can lead to a 70% reduction in harvest index. These results are particularly significant as they challenge the assumption of positive effects of eCO_2_. The literature survey indicates that the buffering effect is conditional and context-dependent. When high temperature coincides with elevated CO_2_, the photosynthetic advantage is not compensated by reproductive failure. From a breeding perspective, it is important to prefer heat tolerance over CO_2_ fertilization effects in future climate scenarios. Combined effect can severely impair soybean yield and yield attributes particularly in presence of weeds. A temperature × CO_2_ interaction has been observed for protein and lipid content in mature soybean seeds, where eCO_2_ with low temperature is beneficial, while with high temperature leads to poorer oil quality [71,73].

The increased atmospheric temperature driven by eCO_2_ intensifies drought stress by amplifying water demand through vapor pressure deficit. Higher humidity and altered precipitation patterns contribute to more frequent, severe, and prolonged droughts through increased evaporation, reduced snowpack, and shifting drought regions. These scenarios pose a risk to cropland productivity if CO_2_ emissions continue to increase, which could destabilize global food security and turn terrestrial ecosystems from carbon sinks into carbon sources [74]. Recent research has highlighted that drought decreases leaf relative water content and turgor pressure, leading to reduction in cell elongation, wilting, and drooping. Stomatal closure leads to limiting CO_2_ uptake, inhibits net photosynthetic rate and carbohydrate synthesis, impairing carbohydrate (and starch) metabolism and transport from source to sink (seeds), significantly leading to reduced seed weight [75]. The oxidative stress causes damage to cellular components (lipids, proteins, and DNA) [76]. Drought also causes changes in root system architecture (RSA) such that increased root length and number to improve water uptake (in tolerant genotypes). Particularly, during early growth, the onset of drought causes significant reduction in nodule number, its dry weight, root-shoot biomass, nitrogen content, C:N ratio, and shoot total nitrogen fixed [77]. Generally, it reduces vegetative growth, shortens plant stature, and narrows leaf size while promoting deeper root growth. Because, the most critical damage occurs during reproductive stages [78,79], the stage-specific losses are notable. Since R1-R6 stages show nearly double yield losses than vegetative stages, water availability become the most critical determinant of yield at flowering. Thus, maintaining reproductive function under water deficit would be a better translational strategy to improve yield. Metabolic impacts include diminished nitrogen fixation, impaired protein biosynthesis, and endoplasmic reticulum dysfunction that accumulates misfolded proteins. Additionally, stomatal closure-induced canopy warming reduces pollen germination by 17%, seed number by 45%, and seed weight by 35%, ultimately compromising seed oil content, nutritional quality, and economic returns [78,79]. Overall, severe drought can reduce soybean yield differently at different growth periods i.e., flowering-podding (FPS/R1-R4; 73–82%), seed-filling stage (PF/R5-F6; 42–48%) [80], early seed filling (R5; ~78%) [81], vegetative stage (V; ~40%) [78,79], seed development stages (R5-R7; 45–88%) [82]. For further details, readers are referred to two very detailed review articles by Rasheed, et al. [83] and Wang, et al. [84], which discuss in detail how drought and water deficit episodes significantly disrupt soybean yield and quality and how plant may adapt to these stresses. Nevertheless, the literature indicates that the earlier the stress occurs during the reproductive stages, the more sensitive is the yield penalty. Thus, breeding and management strategies must consider protecting early reproductive stages.

After drought, flooding is the second most damaging abiotic stress for soybean. A study indicated an increased flood exposure from 2020 to 2100, attributing 21.1% to climate change [85]. For soybean, 6% of the global soybean harvested areas (4 million hectares) are flood affected, causing 4% yield reduction [86]; 17 to 43% during the vegetative growth stage and 50 to 56% during the reproductive stage [87]. While flooding submerges plant partially or completely, its impact on soybean and the resulting yield losses differ from waterlogging. In the latter case, soil pores are filled with water, completely saturating rhizosphere, reducing aeration and causing hypoxia at roots. Short-term waterlogging at full pod and beginning seed stages causes reduced seed weight, pod number, seed number, and 100-seed weight, ultimately leading to yield loss [88]. The projected range of yield penalties by 2050 is 10–20%, indicating need for adapting measures. Soybeans can tolerate short periods of submergences (up to 96 h), however, if submerged for over 4 days, it experiences stand loss, reduced vigor, or even complete crop failure (https://ipcm.wisc.edu/blog/2016/06/assessing-flood-damage-to-soybean-2/#:~:text=Grover%20Shannon%2C%20University%20of%20Missouri,fully%20recover%20from%20flooding%20injury; accessed on 12 April 2026). Under submerged/water-logged conditions, membrane integrity is compromised, ion transport and gas exchange through membranes are disturbed, hormonal biosynthesis and signaling is differentially regulated. Cell organelles/components such as plasma membrane, nucleus, DNA, mitochondria, and endoplasmic reticulum are affected, leading to yield losses [89]. Soybean varieties that can extend tolerance from 96 h to 5–7 days would substantially reduce flood risk in region with short-duration waterlogging events. Whereas, the other strategies to adapt to waterlogging are by forming aerenchyma tissue in the root cortex, adventitious roots, elongated internodes, ROS detoxification, stomatal closure, hormonal regulation, and modified energy metabolism [90].

Climate change exacerbates soil salinity through altered hydrological cycles, rising temperatures and sea-level, which collectively increase salt accumulation while reducing soil fertility, particularly in arid, semi-arid, and coastal regions. Recent models project that drylands in South America, southern Australia, Mexico, the southwestern US, and South Africa face the highest salinity risks. Whereas, secondary threats are emerging in Spain, Morocco, northern Algeria, the Sahara, central India, and parts of Mongolia and China by 2100 under current GHG trajectories. Conversely, regions like the northwestern US, Eastern Europe, and Central Asia may experience stable or reduced salinity. In soybean-growing areas, salinity stress, driven by saltwater intrusion in deltas (e.g., Po River, Nile, Mississippi) and rising water tables in arid zones, severely limits yield by disrupting water uptake, inducing ion toxicity (Na^+^, Cl^−^), and impairing nutrient partitioning [91]. For instance, saline soils reduce water potential, causing osmotic stress that diminishes stomatal conductance, photosynthetic efficiency, and biomass accumulation, while excessive Na^+^ disrupts cellular homeostasis, leading to leaf necrosis and plant mortality in sensitive varieties. Studies demonstrate that 2000 ppm NaCl reduces filled pods per plant by 55–65%, with irrigation timing exacerbating losses (e.g., post-flowering saline irrigation cuts pod numbers by 64.68%) [92]. The wide range in these values reflects not only genotypic variation but also the critical interaction between salinity and irrigation time. Though salinity is dose dependent to an extent, it is also medicated by the developmental stage at which salt stress occurs. This suggests that management strategies may be as important as genetic improvement in mitigating salinity impacts. Thus, breeding programs may incorporate stage-specific screening protocols. Tolerant varieties exhibit adaptive traits such as higher root nodule biomass and sustained leaf greenness under salinity, while others mitigate stress through enhanced transpiration. Mitigation strategies, such as brassinolide foliar application, can ameliorate salinity effects up to 60.60 mM/L NaCl by improving photosynthetic parameters, maintaining K^+^/Na^+^ ratios, and reducing oxidative damage. Brassinolide applied at critical stages (seedling, flowering, podding) significantly restores nutrient partitioning and seed yield [93]. In our assessment, the success of brassinolide application highlights an underutilized strategy. Therefore, combining genetic tolerance with targeted phytohormone-based interventions at critical developmental window may offer a practice near-term solution for salinity-prone regions with longer-term genetic improvement programs underway. To address climate-aggravated salinity, integrated approaches, including salt-tolerant cultivars, biochar amendments, and precision irrigation, are critical. For tidal lands, saturated water cultivation systems and stage-specific irrigation management can reduce yield penalties. Without intervention, salinity stress, intensified by El Niño and droughts, will increasingly threaten global soybean production, necessitating urgent adaptation in vulnerable regions.

### 3.3. eCO_2_ Induced Climate Change Intensifies Biotic Stress and Subsequently Impacts Soybean

The synergistic effects of eCO_2_ and associated climate change factors, including rising temperatures, altered precipitation patterns, and shifting humidity regimes, are fundamentally transforming soybean-pathogen interactions. These changes manifest through three primary mechanisms: (i) expanded geographic ranges of pests and pathogens, (ii) modified host physiology and defense responses, and (iii) accelerated pathogen evolution. This three-part framework provides a conceptual model for anticipating future disease pressures. However, most breeding programs still operate under static geography-specific resistance strategies that do not account for these dynamic shifts. Collectively, the three mechanisms exacerbate biotic stress, with projected yield losses of 11.0–32.4% under climate change scenarios [37,38]. This projected wide range reflects substantial uncertainty in future disease dynamics. Here the lower bound (11%) likely assumes successful adaptation and management, while the upper bound (32.4%) could be worst case scenario with no interventions. Breeders should therefore plan for the higher end of this range to build resilience against wors-case scenario. Climate change driven high temperatures facilitate poleward migration of soybean pests and pathogens. MaxEnt niche modeling predicts that soybean true bug pests will expand into mid-latitude production zones, with Southeast North America, Central South America, Europe, and East Asia facing the highest invasion risks [94]. Similarly, pathogens like *Heterodera glycines* (soybean cyst nematode) are projected to colonize northern regions as temperature ranges for soybean cultivation shift [95]. These projections are of practical importance, as they suggest that historically the genes in southern or tropical may become essential in northern breeding programs within next two-three decades. Thus, pre-emptive introgression of these resistance alleles into northern-adapted germplasm could be initiated. Extreme weather events further modulate disease dynamics, heavy rainfall coupled with warming increases *Phytophthora sojae* (causing soybean root rot) prevalence [96], while drought conditions favor *Macrophomina phaseolina* (charcoal rot) through microsclerotia persistence in soil [97]. This contrasting response to moisture regimes presences a significant breeding challenge. For example, a variety resistant to root rot (favored by wet conditions) may remain susceptible to charcoal rot (favored by dry conditions) and vice versa. This trade-off necessitates region-specific breeding strategies and development of resilient ideotypes that combine tolerance to both wet- and dry-favored pathogens.

eCO_2_ induces complex physiological changes such as stomatal regulation, nutritional dilution, and hormonal cross-talk, that alter soybean susceptibility. Reduced stomatal conductance under eCO_2_ may decrease entry points for stomata-dependent pathogens like *Pseudomonas syringae* pv. glycinea (35–50% reduction observed in analogous systems), though this is counterbalanced by enhanced susceptibility to viruses due to suppressed antiviral defenses [40]. Understanding this dichotomy is critical for breeding as improving resistance for bacterial diseases would improve soybean resilience but viral diseases may become more damaging. Thus, viral resistance development also be given due importance. A 20% decrease in leaf nitrogen and increased C:N ratios under eCO_2_ reduce tissue quality for biotrophic pathogens but may benefit necrotrophs like *Fusarium virguliforme* (SDS). eCO_2_ disrupts the salicylic acid (SA)/jasmonic acid (JA) balance, increasing susceptibility to hemibiotrophs (e.g., *P. sojae*) while enhancing resistance to necrotrophs [98]. Rising CO_2_ and temperatures accelerate pathogen evolution, as demonstrated by increased *Fusarium* aggressiveness under drought stress [99], expanded virulence of *Sclerotinia sclerotiorum* (stem rot) in cooler regions due to warmer winters [100], and reduced efficacy of biocontrol agents like entomopathogenic nematodes under eCO_2_ due to altered root exudate profiles [101]. Together, these instances highlight an overlooked risk: climate change will change the basic biology of diseases, including their host range, aggressiveness, and interactions with biocontrol agents, in addition to changing their distribution. Our key assessment is that more resilient, polygenic resistance techniques may be required since static resistance genes may lose their efficacy more quickly than previously thought. Thus, climate change drives uncertain shifts in pests and pathogens prevalence, altering disease dynamics with altered virulence, and host-pathogen interactions. We contend that breeding programs should take a portfolio approach in light of this uncertainty, combining quantitative (polygenic) resistance with major resistance genes (R-genes), diversifying resistance sources across regions, and keeping sentinel monitoring plots to identify emerging pathotypes before they become epidemic. Breeding for disease resistance, developing accurate forecasting models, strengthening monitoring and surveillance systems, and adapting integrated pest management strategies are key tasks for soybean researchers. Strengthening monitoring and surveillance is, in our opinion, the most important of these, as, in the absence of early discovery of changing pathogen populations, even the finest genetic resistance may be used against the incorrect targets.

### 3.4. Effects of Combined Abiotic Stresses Related to Climate Change on Soybean Yield and Production

**Figure 3:**
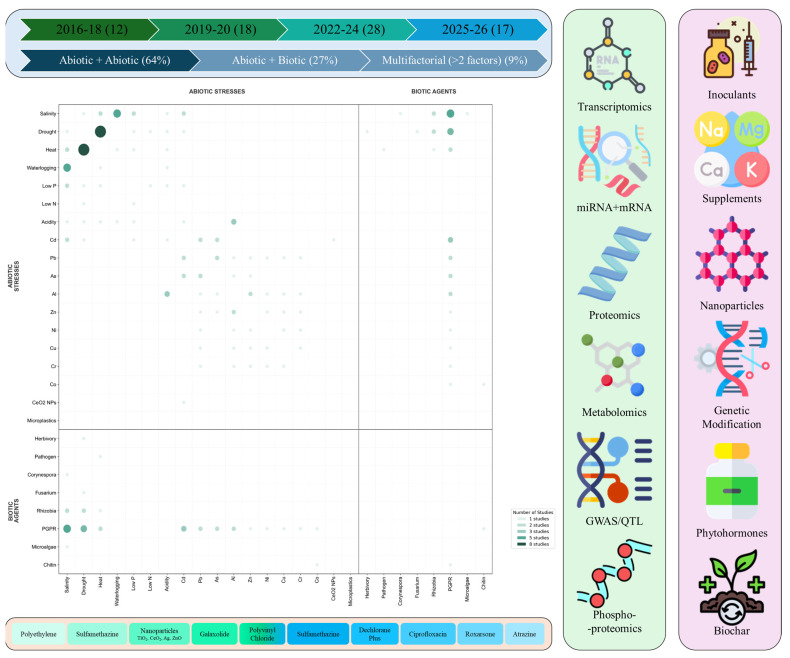
Combined stress research landscape in soybean (An overview of publications retrieved from Pubmed using “soybean,” “Glycine max,” “combined,” and “abiotic stress” as keywords against 2016–2026). Top panels show annual publication trend showing increasing research output together with the distribution of stress combination categories (n = 75 studies). The central heatmap matrix shows which stress-stress combinations were mostly represented in the literature. Circle size and color intensity are proportional to study count. Thick line separates abiotic (top-left) from biotic (bottom-right) stressors. The right panels show technologies employed and mitigation strategies tested for enhancing stress. The bottom panel shows the emerging pollutants represented in the surveyed studies. Figure icons were downloaded from Flaticon (https://www.flaticon.com) and figure was prepared in Microsoft Power Point 2021 Professional Pro.

Major research in soybean, and other plants, has been carried out on the effects of individual abiotic stress on yield and production. However, field conditions are much more complex and the onset of multiple abiotic stressors is common. A systematic survey of PubMed publications (2016–2026) reveals that combined stress research now constitutes a substantial portion of soybean stress biology. Among metal contaminants, cadmium (Cd) emerges as the most critical and versatile stressor, frequently co-occurring with salinity, drought, low phosphorus, soil acidity, and other metals including lead and arsenic. Aluminium (Al) toxicity is intrinsically linked to acidic soils, while chromium (Cr) and cobalt (Co) are typically studied with chemical or biological mitigators rather than in combination with other abiotic stresses. Multi-metal complexes involving Al, Zn, Ni, Cu, Pb, and Cr reflect real-world soil contamination scenarios where multiple metals co-occur. Emerging anthropogenic pollutants, including nano plastics, microplastics, pharmaceuticals (sulfamethazine, ciprofloxacin), and flame retardants (Dechlorane Plus), have garnered increasing attention, often exhibiting dose-dependent and synergistic toxicity when combined with conventional stressors. Technologically, the field has shifted toward multi-omics integration, with transcriptomics serving as the foundational approach, complemented by proteomics, metabolomics, and increasingly, integrated omics platforms. Mitigation strategies have diversified from single-agent applications to combinatorial approaches, with microbial inoculation (PGPR, rhizobia, endophytes) emerging as the most versatile strategy, often combined with nutrient supplements (silicon, selenium, calcium), nanoparticles, or biochar to enhance stress tolerance through synergistic mechanisms (Figure 3).

Recent studies have shown that under controlled environments, combined high-temperature and water stresses or waterlogging reduce soybean yield by 64–91% [102,103]. However, they impact in stage-specific manner (e.g., transition from vegetative to reproductive stages) can impact physiological processes causing failure of yield components [38]. These combinations supress photosynthetic capacity and reduce seed weight and number per plant [38,104].

At the molecular and biochemical level, combined stresses trigger a complex reprogramming of soybean’s transcriptome and metabolome for cellular homeostasis. The stress response is orchestrated by a network of phytohormones, with abscisic acid (ABA) serving as the central regulator [105]. ABA levels surge to mediate stomatal closure and activate stress-responsive pathways, but its signalling engages in intricate crosstalk with JA, SA, ethylene, and strigolactones [106]. For instance, JA is crucial for acclimation to multifactorial stress combinations, while ethylene modulates responses to combined flooding and salinity [107,108]. This hormonal interplay fine-tunes the balance between stress defence and growth arrest. The cellular environment under combined stress features exacerbated oxidative damage, characterized by elevated levels of ROS like superoxide anions and lipid peroxidation markers such as malondialdehyde (MDA). To counteract this, plants upregulate a suite of antioxidant enzymes such as superoxide dismutation (SOD), catalase (CAT), ascorbate peroxidase (APX), and glutathione reductase [107,108]. Moreover, soybeans accumulate compatible osmolytes like proline and glycine betaine [109]. These biochemical responses are underpinned by large-scale transcriptional changes. Transcriptomics studies have identified several stress-responsive genes e.g., homeobox-leucine zipper protein ATHB-12-like, 9-cis-epoxycarotenoid dioxygenase NCED1, low-temperature-induced 65 kDa protein and maturation-associated protein. Major stress-responsive TFs including *AP2-EREBP*, *bHLH*, *MYB*, *HSF*, *C2C2-Dof*, *G2-like*, *NAC*, *WRKY*, *bZIP*, *TCP*, and *zf-HD* has been identified [110]. Additionally, heat shock transcription factors and heat shock proteins (HSPs) are activated to maintain protein homeostasis under heat-drought conditions, while dehydration-responsive element-binding (DREB) factors and the SOS pathway are engaged under drought-salinity combinations to regulate osmo-protectant synthesis and ion (e.g., Na^+^) homeostasis [109].

## 4. Novel Strategies for Soybean Breeding

The future of soybean breeding fundamentally depends on the development and integration of novel, system-level strategies that move beyond trait selection to address the interconnected challenges of climate resilience, resource efficiency, and global food security. Below we discuss the current understanding on key soybean yield related traits, stress tolerance, pathogen dynamics, and WUE. Moreover, we highlight the importance of phenomics and new technologies for soybean improvement.

### 4.1. Deeper Understanding of the Traits of Interest

Despite marked difference during the last six decades (1960–2024), soybean production needs to be increased by approximately 55% by 2050. Foremost strategy to fulfilling this production demand lies in deeper understanding of genetic variation, stress tolerance mechanisms, agronomic and nutritional traits, optimization of biological nitrogen fixation (BNF), and plant architecture under climate change scenario.

**Figure 4:**
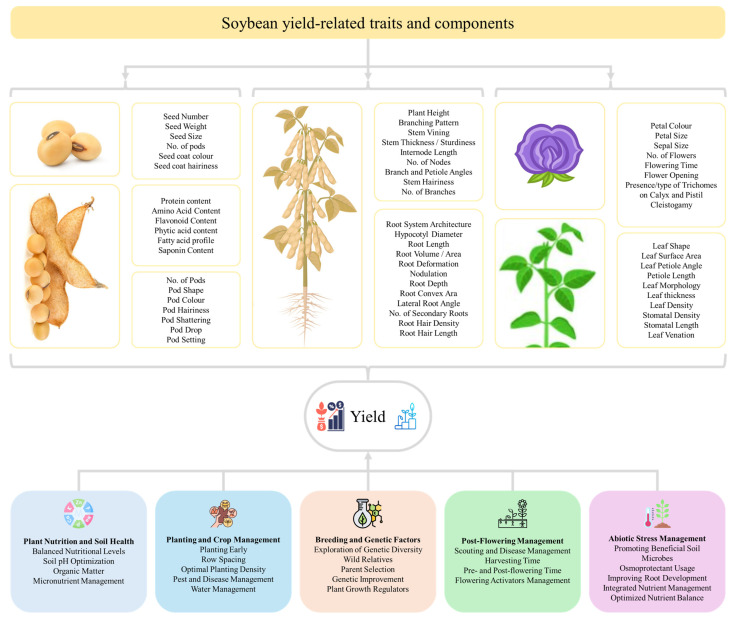
Traits (and components) of interest and strategies for improving soybean yield. The top section of the figure is organized by plant organ and related traits to constitute or effect soybean yield. The below section shows five strategies such as plant nutritional and soil health improvement, planting and crop management, improved breeding and control of genetic factors, post-flowering management, and most importantly, abiotic stress management.

Soybean breeders have been improving multiple aspects of yield (agronomic and nutritional) and adaptation. The achievement of much higher yields [111] than the theoretical maximum yields (see Section 2), indicates that certain growth limiting factors are in play i.e., stresses and agronomic practices. Key yield-contributing traits in soybean are plant height, branching, number of pods per plant, seeds per pod, seed weight, canopy coverage, growth duration. These yield components are severely impacted by abiotic stress, particularly during flowering and pod filling stage. The primary effects of these stresses are reduction in number of pods per plant, number of seeds per pod, followed by decrease in seed weight and size [112]. A critical strategic frontier is to systematically target the genetic networks controlling these yield components to achieve sustainable yield under stress scenarios. To do so, breeders must focus the key traits such as seed number and seed weight, crop growth rate (R1-R5), duration of flowering, leaf area duration, and effective filling period are yield-related components (Figure 4) [113]. Seed number, together with seed size and weight could be a way forward, however, it will be a challenging strategy because the success lies in optimizing source-to-sink relationship as observed in a recent study [12]. Seed size and weight could be targeted, which are governed by complex genetic networks, with over 300 quantitative trait loci (QTLs) mapped for seed weight (e.g., qSw17-1) and fewer for seed size [https://www.soybase.org] [114]. The major genes including *GmCYP78A72* (increases seed size by 7.2%) and *GmKIX8-1* (whose knockout mutants produce 20% heavier seeds via upregulated *GmCYCD3;1-10* expression) can be engineered in existing high-yielding cultivars [115]. A targeted strategy to enhance seed size should prioritize the precise manipulation of key regulatory genes using CRISPR-Cas9 to knockout negative regulators such as ubiquitin protease *GmSW17*, while simultaneously overexpressing the positive effectors like *GmGA3ox1* [116] and *GmST05* (Seed Thickness 05) [117] in elite genetic backgrounds. A complementary strategy would involve developing and employing multi-trait markers to systematically pyramid these modified alleles with favorable native haplotypes, such as CT-repeat variation in *GmWRKY15a* [118]. Nevertheless, seed size is a complex trait and is also linked with the endosperm’s cell division, signalling pathways i.e., ubiquitin-protease route, mitogen-activated protein kinase signalling, transcriptional control, sugar, G protein signalling, HAIKU (IKU) pathways, and plant hormone signalling. However, the overall yield gain from more seeds can outweigh the effect of smaller seed size in many cases [119]. Therefore, key focus of soybean breeders could be to achieve a greater number of seeds per pod, higher number of pods per plant, and increase number of four-seed pods. Particularly, the proportion of seeded-pods should be increased as current varieties set seeds in 50–100 pods against the ability of soybean plants to produce up to 600 pods per plants. This is being achieved by several strategies outlined in Figure 4. Currently, some countries e.g., China have fruitful results in this direction, where CRISPR/Cas9 knockout *Gmjag* (a major locus controlling leaflet shape and seeds per pod) resulted in an increased yield by 8.81% in spring trials [120]. Other strategies to increased pod number can be understanding and manipulation of the pathways contributing towards nutrient allocation to pods e.g., photosystem II-associated gene (*Glyma.10G089300*) and calcium-binding proteins, ABA and calcium signaling [121], and fine-turning florigen expression (increase in 11–24% yield) [122]. Apart from genetics, breeders and agronomists must leverage crucial environmental and agronomic factors modulating soybean pod formation such as photoperiod sensitivity and flower abscission. Compared to short-day conditions, long-day conditions extended the time from flowering to pod formation and led to the abscission of the first wave of flowers. At field level, higher planting density (2.7 × 10^5^ plants ha^−1^) [123], soil pH optimization, nutrient optimization [124], phytohormone application resulted in a 22.8%, 20%, 24%, 3–23.5%, respectively.

Abiotic stresses alter soybean seed chemical composition, primarily affecting the balance of protein, oil, and FAs mainly because of the disruption in photosynthesis and translocation of assimilates during seed filling period [125]. Of the nutritional components, protein and oil are the most economically and nutritionally significant, with typical concentrations ranging from 34–59% protein and 8–28% oil of seed dry weight. Protein concentration generally increases under drought and heat stress (2.3 to 7.1%) but overall protein yield per hectare is significantly reduced [126]. To increase the total seed protein yield and maintain a stable protein concentration, strategies must address yield stability and source-sink balance of nitrogen. For long-term resilience, genetic improvement involving breeding for stable protein lines, protein-oil trade-off, and increased nitrogen fixation efficiency by using genomic and genetic tools, is the key task [127]. Whereas, immediate adaptation strategies can leverage current knowledge on the agronomic management methods such as optimized water management, balanced nutrient management, optimization of sowing dates and cultivar selection, and crop rotation [125]. At the breeding and genetic level, significant progress has been observed with the highest recorded protein+oil content reaching 59% in the “Kruzhnizca” variety, while elite high-protein genotypes like NLM09-77, N14-7017, N16-9924, and Benning HP consistently achieve 50–54% protein under optimal conditions [127,128]. Novel QTLs have also been identified [129,130,131] which showed significant improvement in seed protein stability without protein content penalty. Another successfully strategy to this regard is mining exotic germplasm for genetic improvement of protein quantity and quality in soybean where researchers suggested stacking of protein, cysteine, and methionine QTLs to increase protein and essential oil content [132]. While soybean breeders target improving seed protein for animal feed, human food, and aquaculture industries, this decision comes with established biological and genetic trade-offs. Notably, a negative genetic correlation exists between protein and oil contents, with corresponding adjustments in carbohydrate and fiber levels to maintain total seed mass balance [133]. For example, a higher protein concentration might slightly alter the functional properties of soy flour (e.g., water absorption, texture) in human food processing creating a need to optimize processing methods [134]. Genetic manipulation of protein accumulation can indirectly affect the valuable unsaturated FA percentages (e.g., oleic, linoleic acids). Breeders must monitor this to ensure the resulting oil still meets quality standards for human consumption and industrial uses. As reviewed in detail by Guo, et al. [135], the development of high protein content soybean germplasm will require (i) integration of multiple breeding approaches such as conventional breeding methods with MAS, (ii) use of wild soybean, (iii) use of existing soybean germplasm with higher reported protein contents. To this regard, the authors provided a detailed list of high protein (>45%) content germplasm (see Table 1 in [135]) and several notable examples on the use of wild soybean. Nevertheless, recent developments in genetics, omics, and functional genomics have detailed the mechanism of seed storage protein regulation [136]. Future research at the mechanism level must identify the gene controlling each step of the protein synthesis in seed. Nevertheless, extensive genetic mapping has revealed that seed protein and oil content are complex quantitative traits influenced by numerous loci. Building over the identification of 700 QTLs with 57 confirmed, the way forward for improving seed protein will require precise genetic engineering guided by the characterization of major regulatory genes/QTLs/alleles. Two major QTLs for protein accumulation: cqSeed protein-003 (*Glyma.20G085100* (*POWR1*)) and cqSeed protein-001 (*Glyma.15G049200* (*GmSWEET39*)), provide preferred molecular targets. *POWR1* might be manipulated to enhance nitrogen assimilation and direct it towards protein synthesis [137]. Whereas, *GmSWEET39* activity can be altered to shift carbon flux away from oil biosynthesis and towards amino acids and protein precursor pathways. This might also address protein-oil trade-off [138]. Furthermore, a holistic breeding approach should employ genomic selection models that explicitly weight these major and other minor QTLs such as cqSeed protein-014 (*GmST05*), which interacts with *GmSWEET39* to balance seed composition, and cqSeed protein-013 (which is linked to *Glyma.08G107800* and ST1), influencing both protein/oil ratios and seed morphology. Stacking multiple QTLs will ensure broad-based genetic resilience; a plant with optimized *GmST05*, *GmSWEET39*, and *POWR1* is more likely to maintain stable protein yield across various stresses (drought, heat, variable nitrogen availability) than a plant optimized for just one gene [139]. Such strategies can also be aided by recent knowledge on the transcriptional control of protein content leading to a coordinated genetic improvement strategy considering (i) nitrogen timing and efficiency as *POWR1* likely coordinates circadian clock-associated pathways to optimize nitrogen use for storage proteins (e.g., glycinin, β-conglycinin), (ii) carbon allocation as *GmSWEET39* directs sucrose flux, determining whether carbon skeletons fuel acetyl-CoA (oil precursor) or amino acid (protein precursor) synthesis, and (iii) physical storage capacity (*GmST05*).

However, high protein content in soybean has metabolic trade-offs due to carbon-nitrogen balance; high protein genotypes often exhibit reduced oil, creating a breeding challenge [140]. Thus, strategies to improve seed oil content will differ as compared to those related to protein content and composition. This is because the classical C:N partitioning framework, and the genes operating the biosynthesis of each of the two nutrients, make it challenging to break the correlation. For more detail, readers are referred to the brilliant review by Weiliang et al. [140] to read about the strategies on breaking this negative correlation. In light of climate-change, soybean breeders must focus on making to oil synthesis machinery robust enough to function optimally under suboptimal conditions since abiotic stresses reduce seed oil content e.g., drought can reduce as much as 35% oil content during seed filling stage [141]. Like other traits, the strategies to improve oil content under climate change scenario include genetic methods and optimized agronomic management. Of these, genetic modification is fundamental to increase oil content. It would involve push (increase carbon flux), pull (efficient assembly of triglycerides), package (lipid droplet formation), and protect (minimize triglyceride turnover) strategies [142]. Detailed studies over past few decades have improved our understanding on the metabolic pathways and transcriptional networks of oil accumulation in soybean seeds. To increase carbon flux (push), overexpression of master transcriptional regulators and rate-limiting enzymes would beneficial e.g., genes such as LEAFY COTYLEDON (LEC), *LEC1* and *LEC2*, which orchestrate seed development and storage reserve allocation [143,144]. *LEC1*, an atypical NF-YB subunit, forms a complex with *NF-YA/NF-YC* to activate FA biosynthesis genes, and its overexpression in soybean (*GmLEC1a/b*) enhances FA production by regulating downstream targets like *GmZF351* and *GmZF392* [145]. *LEC2*, a B3 domain TF, synergizes with *LEC1* to upregulate *WRINKLED1 (WRI1)*, a master regulator of triacylglycerol synthesis [146]. In soybean, *GmWRI1a/b* orthologs directly enhance oil content by activating lipid biosynthesis genes, while their knockdown suppresses lipid production, making them prime targets for genetic improvement [147]. Furthermore, overexpression of acetyl-CoA carboxylases and FA export from plastids via fatty acid proteins can be achieved [148,149]. Concurrently, the “pull” strategy would involve efficient triacylglycerol assembly in endoplasmic reticulum by modifying final acylation step. Overexpression of *GmDGAT1A* and *GmDGAT1B* resulted in a 16% relative increase in oil contents in Arabidopsis seeds [150] and within soybean *GmDGAT1b* isoforms can lead to substantial differences in oil accumulation [151]. A critical complementary approach is the “package” strategy, which optimizes lipid droplet formation and stability to prevent lipotoxicity and enable efficient storage. For this, genes such as *GmOLEO1*, an oleosin gene that stabilizes lipid droplets [152], are excellent targets. Overexpression of oleosin and seipin genes has proven effective, with soybean oleosin increasing oil by up to 10.6% in soybean and 46% in rice [152,153]. Finally, the “protect” strategy minimizes triacylglycerol turnover during seed maturation and germination by downregulating lipase activity. Silencing the *SUGAR DEPENDENT1 (SDP1)* lipase increased total soybean seed oil by up to 30% [154]. Whereas, suppressing other lipases like *GDSL1* also boosted oil content in Brassicas [155]. *GDSL1* homologs in soybean can be targeted to achieve similar results. Beyond these core metabolic engineering targets, precision breeding for specific fatty acid profiles is a focused strategy for nutritional and industrial applications. This can be achieved through targeted disruption of desaturase genes using CRISPR-Cas9; for instance, knockout of *FAD2-1A* and *FAD2-1B* elevates oleic acid to over 80% [156], while editing *FAD3* genes (*FAD3A*, *FAD3B*, *FAD3C*) reduces linolenic acid [157]. Similarly, to reduce saturated FAs (up to 53% lower palmitic acid content) can be achieved by knocking out *FATB1a* and *FATB1b* [158]. Whereas, to overcome the negative correlation between oil and protein content, upstream carbon partitioning can be manipulated such as tandem zinc finger proteins *GmZF392* and *GmZF351,* which form a positive feedback loop under *GmLEC1* regulation to amplify oil accumulation [159], and transcription factors like *GmbZIP123* [160], which modulates sugar transport to boost lipid synthesis. Furthermore, modifying sugar transporters like *GmSWEET10a* and *GmSWEET10b*, which control sucrose delivery to the embryo, can shift resource allocation [161], while engineering malic enzyme activity can direct glutamine-derived carbon toward FA synthesis [162]. Nevertheless, the integration of multi-omics data with advanced genome editing tools like CRISPR-Cas9, base editors, and prime editors can enable the precise stacking of these favorable “push,” “pull,” “package,” “protect,” and composition-modifying alleles. This synergistic approach allows for the solving the problems of metabolic trade-offs and the *de novo* design of soybean genotypes with optimized oil yield and preferred FA composition to meet specific nutritional and industrial demands. Besides, it is important to note that the United States Department of Agriculture reports average compositions of 36.49% protein, 19.94% oil (of which 11.2% is unsaturated fatty acids), 30.16% carbohydrates, and 9.3% fiber, highlighting soybean’s role as a balanced nutrient source [143]. Therefore, breeding for a balanced composition should prioritize several factors including stability, nutrient density, and overcoming negative genetic correlations, by using advanced genomic tools and exotic germplasm.

While increasing seed quality and quantity remains a primary objective of soybean breeders, other traits exert a direct influence on yield potential by influencing vegetative growth and reproductive efficiency. To better exploit hybrid-vigour, a key biotechnological strategy involves re-engineering soybean flowers to make them more visible to pollinators with sweater nectar. However, efforts based on the CRISPR-based activation *GmANT* and repression of *GmBPE* resulted in reduced petal size [163], indicating a complex regulatory network governing petal development. Though, recently developed, high-resolution phenological map of flower development in soybean [164], together with the GWAS based genetic map for petal size variation [165] can help breeder to pinpoint role of key genes in petal size and cell expansion and provide prime target genes for manipulation. Apart from floral biology and architecture, the knowledge on the genetic control of flower pigmentation [166] can be integrated to select or engineer specific pigmentation alleles to optimize pollinator attraction. However, modifying flower architecture is a challenging strategy considering limited role of cross-pollination in field conditions [163]. Moreover, abiotic stresses majorly affect flower viability and pollen function, therefore, future breeding may prioritize addressing these challenges. In this regard, screening of parents exhibiting better pollen germination rates [167], engineering genes to maintain higher ABA levels in reproductive tissues (like in tomato) [168], and overexpressing heat shock proteins to improve male fertility [169] under stress scenario are key directions. Climate change driven weather shifts may disrupt the fine-tuned photoperiod in soybean. The development of early maturing cultivars for high latitudes (by manipulating E1 gene) [170] and for low-latitudes (by manipulating J gene) [171], highlights how understanding and manipulating the genetic control of photoperiod response in fundamental to soybean’s expansion. Soybean breeders must prioritize to combine multiple favourable alleles (*e1*, *e2*, *e3*, *e4*, *J*) and *FLOWERING LOCUS T 5b* [172] to breed cultivars with broad adaptability across various climates [173].

Other than seeds and flowers, the above and below ground architecture of soybean plants are also linked with plant productivity and grain yield. Shoot architecture is correlated with light interception, photosynthetic efficiency, response to agronomic inputs, and adaptation to changing environment [174]. High temperatures and drought, alone or combined, impair canopy architecture, reduce biomass and height, and later root-to-shoot ratio, leading to yield losses [80]. Strategic improvement of soybean canopy and shoot architecture for climate resilience requires a multi-trait, systems-based approach targeting a core genetic element to optimize light interception, lodging resistance, and resource use efficiency under stress. The foundational strategy is engineering an optimal stem growth habit by manipulating the *Dt1-GmFT5a* regulatory module. Introducing loss-of-function dt1 alleles or using CRISPR/Cas9 to edit *Dt1* would induce determinacy for shorter stature and synchronized maturity [175], which will be ideal for stable environments. For greater plasticity under variable conditions, targeting the *Dt2* locus to develop semideterminate varieties [176] offers a superior compromise, balancing continued vegetative growth with terminal flowering to buffer yield against terminal drought or heat. At the same time, plant height and lodging resistance must be engineered by manipulating the gibberellin (GA) and brassinosteroid (BR) pathways. Key targets include *GmDW1* (GA biosynthesis) [177] and *GmDWF1* (BR biosynthesis) [178] for reduced internode length, as well as *GmLHY* and PH13/PHP which integrate circadian and light signalling to suppress shade-avoidance elongation under high-density planting [179,180]. Higher node number can be achieved by delaying floral transition by manipulating *GmFT2a* or by releasing apical dominance by knocking out the branching repressors (*GmSPL9* family and *GmBRC1*) or overexpressing *GmmiR156b* [181,182]. Canopy photosynthetic efficiency can also be optimized by modifying leaf architecture. For example, editing *GmJAG1* narrows leaflets for better light penetration and higher seed set per pod [120], while modulating *GmILPA1* or *GmPIN1* reduces petiole angles for an upright canopy [183]. The forward strategy under climate change must leverage CRISPR/Cas9 system to pyramid these alleles, stacking semideterminacy (*Dt2*), dwarfism (*GmDW1/GmDWF1*), high branching (*gmspl9*), and erect leaf (*gmilpa1*) traits, into a single, climate-resilient ideotype. This ideotype must be validated using high-throughput phenomics and growth models across stress gradients to ensure the engineered architecture maintains yield stability under drought, heat, and high-density stress. Ultimately, the integration of this precision breeding with adaptive management, such as tailored planting densities, will be essential to deploy compact, high-yielding soybeans capable of sustaining productivity in a variable climate. While we highlight major genes related to these traits, other genes, alleles, TFs, and miRNAs playing role in architecture are also valuable targets, for which the readers are referred to recently published detailed reviews [174,181,182].

For below ground organs, RSA in soybean is primarily shaped by genetic, hormonal, and environmental factors that collectively influence root growth, branching, and nutrient acquisition efficiency. Abiotic stress alters root morphology and function, where roots exhibit plasticity and adapt to maximize water and nutrient uptake, however, it costs overall biomass and yield reduction [184]. Recent studies have shown that drought induces increased root length and steeper primary root growth together with inhibition of ethylene synthesis, ABA biosynthesis, reduced water loss, and disturbed nodulation [185]. Whereas, high temperature reduces cell division, inhibiting root elongation, and overall root biomass, leading to reduced flower and seed set [186]. Salinity disrupts ion transport with excessive Na^+^ accumulation impeding nutrient absorption and causing suppressed plant growth [187]. Broadly speaking, RSA is strategically determined by genetic mechanisms that enable plant to sense and integrate environmental signals through auxin-regulated circuits [188]. To breed soybean with climate-resilient superior RSA, breeders must target specific, advantageous root ideotypes by manipulating the system’s key genetic determinants; gravitropic set-point angle (GSA) and lateral root initiation and branching (LRB), while integrating the knowledge on cellular, hormonal, and environmental regulation. At the cellular level, enhancing the transient but critical root hairs in order to continuously expand the absorptive surface area is required [184]. To this regard, the expression of key genes regulating root development, including β-expansins (*GmEXPA1* and *GmEXPA2*) that promote cell division and elongation can be modulated [189]. Precision engineering of GSA regulatory module can help achieving required root depth by deploying MAS or CRISPR-Cas9 to introgress favorable *EXOCYST70A3* haplotypes. These haplotypes have been identified in Arabidopsis for controlling deep (haplogroup H) or shallow (haplogroup C) rooting [190]. At the same time, editing soybean homologs of auxin transporter genes (e.g., *AtPIN4*) can stabilize a desired GSA [190,191], thereby creating heritably deeper root systems for drought resilience or shallower, fibrous systems for efficient topsoil foraging. Whereas, enhancing LRB density and hydropatterning efficiency requires identification and stacking of high LRB QTLs (e.g., LRN locus on Chr 16 [192]) via MAS. Similarly, the existing knowledge on the auxin-response network in Arabidopsis can be leveraged to fine-tune it in soybean as the key components of the network e.g., ARFs [193], IAAS [194], and SUMO [195] family members have been discovered in soybean. This would improve branching in wet zones and suppress in dry soils, thereby eliminating wasteful carbon expenditure. This is regulated by hormonal cross-talk, where ABA (involving molybdenum cofactor sulphurase (*LOS5/ABA3*) [184]) increases root hydraulic conductivity and water uptake, while ethylene interacts with ABA to fine-tune root elongation and lateral root initiation [196,197]. Beyond core developmental genes, shoot-root signaling pathways (e.g., *CEP-CEPR1* in Medicago [198] or *GmCEP6* [199]) can be manipulated (edited/overexpressed) to optimize systemic resource allocation under nitrogen limitation, ensuring roots adjust their branching patterns to exploit localized nutrient-rich zones. Furthermore, the genetic targets [200], available root phenotypic plasticity in soybean germplasm [201,202], together with high-throughput 3D phenotyping to classify root ideotypes (e.g., “steep, deep, and cheap” taproots vs. shallow-fibrous systems) can be leveraged for RSA improvement. Finally, these genetic gains must be integrated with adaptive agronomic practices (e.g., strategic phosphorus banding to guide LR proliferation [203]) and microbiome engineering, such as inoculation with beneficial microbes like *Bacillus subtilis*, which consistently enhances root length, surface area, volume, and biomass [204,205,206]. The ultimate objective is to develop a carbon efficient yet highly plastic RSA ideotype that dynamically optimizes soil exploration per unit of carbon cost, ensuring stable water and nutrient uptake under the increasing variability of future climates.

**Figure 5:**
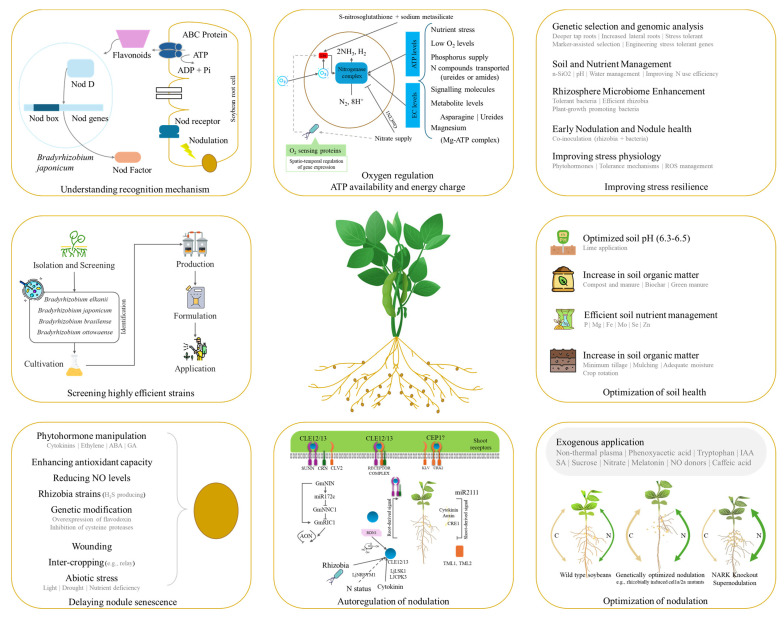
Strategies to improve nitrogen fixation in soybean. The recognition mechanisms by which soybean recognizes rhizobia and the autoregulation of nodulation pathway need improved understanding. Meanwhile isolation and screening of highly efficient rhizobia strains can lead towards development of novel formulations and large-scale application. Nodulation can be optimized by using rhizobially induced cle1a/2a (ric1a/2a) and exogenous application of non-thermal plasma, phenoxyacetic acid, tryptophan, Indole-3-acetic acid, salicylic acid, sucrose, nitrate, melatonin, NO donors, and caffeic acid. Apart from numbers, early nodulation, nodule health, and delaying nodule senescence are way forward. Moreover, the oxygen regulation, ATP availability and energy change in nodules should be further understood. Under climate-change scenario, improvement of stress resilience by rhizosphere enhancement, soil and nutrient management, genetic selection and genomic analysis of root and tolerance traits, improving early stress physiology. Soil health management improves nitrogen fixation by optimizing soil pH, increase in soil organic matter, efficient nutrient management, and soil structure improvement. Figure panel on the autoregulation of nodulation was adapted and redesigned from [215]. Figure panel on optimization of nodulation was adapted and redesigned from [216]. Figure was prepared in Microsoft Office Power Point Professional Plus 2021.

Another below ground feature of soybeans is nitrogen fixation. Climate change-driven stresses impair nodule formation and alter the source-sink balance of carbon and nitrogen causing up to 80% reduced N fixation leading to 40–90% lower yields, especially during seed filling stages [207]. A primary goal is to enhance symbiotic nitrogen fixation by improving nodulation efficiency, nitrogenase activity, and stress resilience. This can be achieved by understanding the recognition mechanism between rhizobia and soybean to expand host range of rhizobia infection, screening highly efficient strains, delaying nodule senescence, and solving problems of nodulation inefficiency under abiotic stress to breed and engineer stress-tolerant soybean cultivars and rhizobia strains. To improve the host plant, soybean can be genetically engineered to optimize carbon-nitrogen trade-off and build stress resilience directly into the symbiosis. Key strategies must include (1) fine-tuning autoregulation to moderately increase nodule number without yield penalty (e.g., *RIC1a/2a* mutation); (2) decoupling nodulation from nitrate inhibition via editing of *GmNLP1/NLP4* for BNF persistence in high-N soils; (3) delaying nodule senescence to extend the functional N-fixing period (e.g., knockout of cysteine proteases); (4) enhancing intrinsic stress tolerance of nodules through overexpression of chaperones (*GmNOD100* for heat) and leghaemoglobins; and (5) securing carbon allocation to nodules under stress by modifying sugar transporters (*GmSWEET10a/b*) (Table 2). For microbial symbiont enhancement, superior rhizobia strains can be selected or engineered to overcome specific climate stressors. Key approaches include: developing cytokinin-overproducing or osmoprotectant-synthesizing rhizobia to improve nodulation under water deficit; selecting heat-tolerant strains expressing chaperones like ClpB (e.g., in *Sinorhizobium*) or cold-tolerant strains to maintain function under temperature extremes; and utilizing acid-tolerant rhizobia for nodulation in low-pH soils [208]. A highly effective approach is the dual inoculation of rhizobia with free-living, high-ACC deaminase-producing plant growth-promoting bacteria (e.g., *Pseudomonas* spp. strains OFT2/OFT5), which degrades the ethylene precursor ACC to mitigate drought- and salinity-induced nodulation suppression [209]. Furthermore, engineering nitrate-tolerant rhizobia strains allows for efficient nodulation even in nitrogen-rich soils [210], and the use of Nod factor-enriched inoculants (e.g., as used in pea and vetch [211]) can overcome impaired signal diffusion in dry soils to ensure robust symbiosis establishment. Research has shown that integrated adaptive management can help realizing genetic gains. For example, the number and size of nodules can also be increased by enhancing nitrogenase activity by seed treatment with non-thermal plasmas [212], nickel, Co, molybdenum [213], biochar and biofertilizer input [214], and foliar application of nitrogen-fixing agents. Collectively, by integrating precise soybean genetics, resilient microbial consortia, and optimized agronomic practices, soybean production can achieve efficient, climate-resilient nitrogen fixation, ensuring yield stability with a reduced environmental footprint (Figure 5).

**Table 2:** Summary of key genes that have been modified to improve nodulation in soybean.

Gene(s) Modified (Type of Modification)	Effect on BNF/Nodulation	Impact on Yield & Related Traits
Autoregulation of nodulation (AON) pathway		
*RIC1a/2a* (Rhizobia-Induced CLE 1a/2a)—(KO/CRISPR)	Moderately increased nodule number; improved N & P content; enhanced carbon/nitrogen balance.	10–31% increase in grain yield and increased protein content in field trials [216].
*NARK* (Nodule Autoregulation Receptor Kinase)—(KO/Mutation)	Supernodulation (excessive nodule formation).	Often results in a yield penalty or stunted shoot growth due to excessive carbon drain [217].
Nitrate inhibition of nodulation		
*GmNIC1a/b* (Nitrate-Induced CLE 1a/b)—(OE)	Inhibited nodulation in a *GmNARK*-dependent manner.	Not specified [218].
*GmNLP1*, *GmNLP4* (NIN-like Proteins)—(KO/CRISPR)	Unveiled a nitrate-tolerant nodulation phenotype, allowing nodulation even with high nitrate.	Not specified [219].
Phosphate (Pi) homeostasis & nutrient acquisition		
*GmPHR1* (PHOSPHATE-STARVATION-RESPONSE 1)—(OE)	Promotes nodulation, increases nodule size, enhances N & P acquisition, boosts nitrogenase activity.	~10.8% increase in single-plant yield under field conditions [220].
*GmPAP4* (Acid Phosphatase)—(Underexpression)	Significantly affected nodulation and BNF efficiency under phosphorus-deficient conditions.	Affected yield under P-deficient conditions [221].
*GmEXPB2* (β-expansin)—(OE)	Enhanced nodule enlargement, increased infected cell abundance, improved N2 fixation capacity.	Promoted increases in N and P content, biomass, and yield under low P stress [222].
Nodule senescence		
*GmCYP35*, *GmCYP37*, *GmCYP39*, *GmCYP45* (Cysteine Proteases)—(Quadruple KO)	Delayed nodule senescence, significantly higher nitrogenase activity in older nodules.	Potential for prolonged N-fixing period (field impact not specified) [223].
*GmNAC039*, *GmNAC018* (NAC Transcription Factors)—(OE/Mutation)	Overexpression causes early senescence; mutants delay senescence.	Not specified [223].
Plant hormone signaling		
*GmCRE1* (Cytokinin Response 1)—(KO)	Decreased nodule number and size, almost complete abrogation of BNF.	Significant yield reduction [224].
*GmRR11d* (B-type Response Regulator)—(KO/CRISPR)	Significantly increased nodule numbers (nearly doubled in hairy roots).	Not specified in current field studies [225].
Gibberellin receptor gene—(Editing)	Improves N fixation and overall plant growth processes.	Improves yield [226].
*GmNMH7* (MADS-box TF)—(OE)	Inhibited root and nodule development, acts as a negative regulator.	Not specified [227].
Nodule organogenesis (central regulator)		
*GmNINa* (Nodule Inception a)—(OE/Knockdown)	Central regulator; required for nodule formation. Knockdown inhibits root hair deformation and infection threads.	Not specified [228].
Carbon metabolism and allocation		
*GmSWEET10a/b*—*(OE/KO)*, *GmSWEET3c*, *GmSWEET38*	Regulates sugar transport to seeds/nodules. OE increases sugar availability to nodules.	OE increases single-plant yield by 11–20%; KO decreases yield by 40.2% [229].
Nodule function (micronutrient cofactors)		
Leghaemoglobin (*Lb* genes)—(OE)	Regulates internal oxygen levels to protect nitrogenase from oxidative damage.	Not specified [230].
Root nodule symbiotic specificity		
*Rj4* (Thaumatin-Like Protein)—(Mutation)	Controls nodulation specificity; recessive mutant allows nodulation with otherwise incompatible strains.	Critical for managing specific rhizobial inoculants (not a direct yield trait) [231].

Abbreviations: KO = Knockout, OE = Overexpression, CRISPR = CRISPR-Cas9 mediated editing, TF = Transcription Factor.

### 4.2. Understanding Soybean’s (Abiotic) Stress Physiology

Understanding the physiological mechanisms underlying soybean’s adaptation and tolerance to abiotic stresses is essential for developing climate-resilient varieties. Soybean employs unique strategies to maintain productivity under adverse conditions, integrating rapid acclimation with long-term adaptive traits. Detailed research on soybean has catalogued these mechanisms that can enable soybean breeders to develop a strategic research and breeding process [232]. This process must prioritize which mechanisms are most amenable to manipulation for synergistic multi-stress resilience. Soybean perceives abiotic stresses through sensing mechanisms, including osmotic and ionic imbalances, ROS signaling, and hormonal regulation. Drought and salinity disrupt water uptake, triggering osmotic stress sensors that activate adaptive responses such as stomatal closure mediated by ABA. Concurrently, stress-induced ROS act as secondary messengers, modulating gene expression while posing oxidative risks if unchecked. Hormonal cross-talk, particularly involving ABA, ethylene, and JA, fine-tunes these responses, with calcium signaling further integrating stress cues into metabolic adjustments [233]. Breeders should leverage this foundational knowledge by targeting key genes in the signaling network (e.g., specific Ca^2+^ sensors or hub transcription factors) for genetic enhancement, rather than treating each pathway in isolation. Upon exposure to stress, soybean experiences rapid physiological disruptions, including membrane dysfunction, photosynthetic inhibition, and energy deficits. The plasma membrane’s integrity is compromised, leading to altered fluidity and electrolyte leakage. Photosynthesis declines due to stomatal closure limiting CO_2_ uptake, while mitochondrial dysfunction reduces ATP availability. Nutrient imbalances, particularly under salinity, impair ion homeostasis and nitrogen fixation, compounding yield losses [234]. Therefore, a core strategic pillar must be the development of integrated phenotyping platforms to quantify these disruptions in real-time, linking them to molecular markers to accelerate the selection of genotypes with inherently stable membrane composition, photoprotective capacity, and nitrogen fixation under stress. To mitigate these effects, soybean employs short-term acclimation strategies, such as osmoprotectant accumulation and antioxidant defense activation. Long-term adaptation involves genetic and epigenetic modifications, with breeding programs targeting traits like drought-tolerant root architectures and salt-exclusion mechanisms. Future breeding programs must move beyond targeting individual traits to a systems-based strategy: aiming pyramiding alleles for short-term acclimation (e.g., antioxidant efficiency) with those for long-term structural adaptation (e.g., deep rooting), using genomic prediction models to identify optimal allelic combinations for target environments. Below, we discuss the morphological, physiological, biochemical, and metabolic adaptations that enable soybean to withstand abiotic stresses, providing a comprehensive framework for improving climate resilience.

At morphological level, soybean’s responses under abiotic stress leads towards survival through alterations in RSA, canopy temperature regulations, and leaf morphology. These responses are dependent on stress type, climate regimes, growth stages, duration, and their interactions. Under drought, the soybean root system typically shifts from lateral root proliferation to developing axial roots, increase root length and root-shoot ratio in order to access deeper water sources. Meanwhile, lateral root density and diameter are reduced [235]. Under waterlogging, soybean plant develops adventitious/aerial roots and form aerenchyma for oxygen transport. Salinity triggers a negative halotropic response, often leading to reduced root length and biomass, though some genotypes increase root-to-shoot ratio or form shallower angles. Temperature extremes promote the formation of higher-order lateral roots to compensate for reduced uptake efficiency [236]. By defining ideal root ideotype (deep taproot, increased length, and density at deeper soil layers [237]) breeders can guide MAS process by the known QTLs [238] and genes [200], and improved high-throughput phenotyping (HTP) platforms [239]. At genetic level, superior haplotypes of key genes e.g., DEEP ROOTING 1 homolog, *GmGA20ox1*, *GmEXPB2*, and *GmPTF1*, *GmPHR1*, etc., can be developed into selection markers for root growth angle, primary root length, root biomass accumulation, phosphate deficiency, and lateral root development, respectively [220,240]. Breeders must focus on identifying stress specific genotype selection and inclusion in breeding programs. For example, genotypes with rapid and pronounced ethylene-mediated responses such as flood-tolerant accessions (e.g., PI408105A), show earlier and stronger induction of ethylene biosynthesis pathway genes, leading to quicker adventitious root and aerenchyma formation, critical traits for oxygen transport and post-submergence recovery [241]. Similarly, salinity resilience requires a dual strategy i.e., selecting for the negative halotropic responses to encourage avoidance of saline patches, and ensuring robust ion homeostasis. The knowledge gained at the genetic regulation of these traits as well as general stress responses in soybean can be leverage for genetic improvement. To this regard, several brilliant reviews on the salt tolerance [242], drought [83], water stress [184], and heat stress [64] can be consulted for extended gene lists. In addition to the key genes, extended knowledge on the genetic regulators (including TFs [243]) for enhanced nutrient acquisition under limiting conditions are essential for climate resilience [220]. Nevertheless, translating this genetic knowledge into climate-resilient cultivars requires an integrated breeding pipeline. The defined root ideotypes must be selected using HTP platforms capable of quantifying root growth angle, depth, and aerenchyma formation non-destructively, and linked with GS models that incorporate the major QTLs and candidate genes. Ultimately, the strategic pyramidion of these alleles, deep rooting (DRO1, *GmGA20ox1*), waterlogging tolerance (ethylene-responsive traits), halotropic avoidance (qSOS1), and enhanced nutrient foraging (*GmPHR1*, *GmPTF1-GmEXPB2*), through MAS and gene editing will create soybean varieties with root systems pre-adapted to the complex, interactive stresses of future climates, ensuring stable water and nutrient acquisition and yield security.

The remarkable morphological plasticity of above-ground soybean organs is well documented [83,244]. The transition from understanding the morphology to engineering it defines the future of climate-smart soybean breeding with a focus on integrating traits for efficiency, avoidance, and recovery into an ideal plant architecture. Canopy temperature dynamics and leaf architecture serve as important indicators of stress tolerance [103]. Leaf growth orientation, controlled by petiole angle relative to the main stem, significantly influences photosynthetic efficiency and canopy microclimate. When challenged with drought stress, soybean leaves undergo multiple protective modifications: reduced surface area minimizes transpiration; rolling/flipping decreases solar exposure; stomatal closure conserves water; and altered mesophyll cell structure improves water retention [245]. Heat stress induces complementary adaptations including leaf thinning for better heat dissipation and chloroplast structural adjustments to maintain photosynthetic function. Some genotypes develop thicker cuticles or increased trichome density to reflect radiation and conserve water [246]. The future soybean varieties should have compact and efficient canopy ideotype by optimizing light interception and microclimate under high-density planting. Key genetic elements, natural or induced variations e.g., rin1 (SPA3a) conferring short internodes [247], mutation breeding (180 registered mutant varieties; https://nucleus.iaea.org/sites/mvd; accessed on 5 December 2025), selection of erect leaf habit modulated by genes like PIN1a/c and ILPA1, together with agronomic strategies (e.g., narrow spacing [248]), have already been explored. Selection of such ideotypes can also be accelerated by HTP. Simultaneously, water responsive genotypes with cooler leaves (canopy) can tolerate drought. Slow-wilting trait can be introgressed to maintain cooler canopy e.g., PI416937 with restricted transpiration under high vapor pressure [249]. This trait can be combined with alleles for specific leaf area from cultivars like G4620RX [167]. While the ideal morphology can be engineering or selected through MAS, it is also important to exploit temporal plasticity through life cycle manipulation. This will ensure avoiding terminal stresses (drought and heat). It can be achieved agronomically through early soybean production systems and genetically by selecting early maturity. This strategy can be optimized using predictive crop growth simulation models like DSSAT for future climate scenarios [250]. Overall soybean plant’s morphological improvement has also been strategically achieved by seed priming and chemical treatments e.g., silicon application, PGPMs, nanoparticles together with specific rhizobia [251]. This synthesis of morphology and management, guided by advanced phenotyping, molecular tools, and predictive modeling, constitutes the core strategy for breeding a climate-resilient soybean crop. These morphological changes integrate with physiological and biochemical responses through ROS-hormonal crosstalk [246].

To develop soybean varieties for climate change and stress scenarios, it is important to understand how different stress impact soybean at physiological and biochemical scale. Generally, like other plants, soybean is significantly impacted by abiotic stresses through direct effects on photosynthetic and respiratory processes. Key physiological indicators of stress response include chlorophyll fluorescence parameters such as performance index on absorption basis, reaction centers per cross-section, that correlate with relative water content and oxidative damage. While these are key indicators, soybean breeders can adopt them as early-stage screening tools. For example, Kokebie, et al. [252] used chlorophyll fluorescence (Fv/Fm) and photosynthetic pigments as indicators of salt tolerance. Rubisco abundance declines during prolonged drought, limiting carboxylation efficiency, which can allow breeders to shift from general yield-based selection to targeted physiological breeding. To this regard, the antioxidant defence system can also be used as a primary selection criterion for climate-resilient soybean germplasm. Moreover, the knowledge that superior genotypes exhibit induction of SOD exceeding an 80% increase, accumulation of proline and sugars (osmotic adjustment), enhanced antioxidant activity, maintaining relative water content (RWC) above 70%, leaf water potential [253], increased MDA levels (up to 111%) [254], and cell membrane stability, provide quantifiable biochemical targets [76]. These targets can be leveraged for identifying breeding lines capable of mitigating oxidative damage. These physiological responses are also visible under salinity stress, which disrupts ion homeostasis, leading to Na^+^ toxicity and osmotic imbalance. Soybean regulates Na^+^ uptake through selective transporters including Na^+^/H^+^ antiporters, compartmentalizing excess Na^+^ in vacuoles [255]. Therefore, development of molecular markers based on ion transports e.g., *GmSALT3* [256] are ideal tools for salt stress tolerance breeding. Another strategy could be monitoring SPAD values to screen for nitrogen efficiency; genotypes that limit chlorophyll degradation to less than 15% (avoiding the typical 35% decline) demonstrate superior chloroplast protection and should be favored for saline-alkali soil adaptation [257]. In many soils, Cl^−^ toxicity is as damaging as Na^+^, therefore, selection for *GmCHX* transporters can help in excluding Cl^−^ from leaf tissue, making it a critical but often overlooked marker for long-term canopy health [258]. Among key physiological targets, K^+^/Na^+^ ratio (above specific thresholds e.g., genotypes with >1.0 ratio in leaf tissue demonstrate superior cellular homeostasis) can be used as a definitive marker to distinguish salt tolerant and sensitive cultivars [259]. Another key physiological change in soybean is hypoxia in response to waterlogging. In this case, breeding programs must prioritize genotypes that demonstrate metabolic shift such as alanine accumulation rather than ethanol production, better recovery by maintaining enough photosynthetic machinery, and ATP conservation efficiency [260]. Under combined drought and heat stress, complex physiological responses occur: stomatal closure conserves water but limits transpirational cooling, increasing leaf temperature 1–2 °C compared to heat stress alone [261]. Breeders must select varieties exhibiting fast recovery involving stomatal reopening and restoration of gas exchange, as degradation of ABA and metabolic cleanup of ROS and prevent secondary oxidative burst, though prolonged hypoxia causes irreversible damage to photosystem II proteins [262]. Sakukei 4 (a Japanese variety) showed better ability to remobilize nitrogen via enhanced nodule growth. Similarly, the famous Williams 82 is often used to demonstrate a rapid transcriptomic and physiological reset of the TCA cycle for new vegetative growth [263,264].

Available data on soybean indicates that it employs coordinated biochemical responses and triggers the accumulation of important molecules i.e., soluble sugars, amino acids, flavonoids, proline, glycine betaine, sucrose, fructose. By understanding these responses for specific stress type, growth and developmental stage, stress duration, and genotype, we can identify and employ specific biochemical markers to be used in breeding programs [265]. For example, under drought, the increased proline accumulation in tolerant genotypes via the glutamate pathway, and concurrent reduction in MDA content through ROS scavenging provide prime targets for genetic enhancement. For this, recent advances have focused on manipulating proline biosynthesis pathway to increase its endogenous levels by overexpressing *GmDREB2* [266] and *GmDREB6* TF [267] that may lead to increased Δ1-pyrroline-5-carboxylate synthetase (P5CS) expression, or targeting proline dehydrogenase (ProDH) to reduce proline degradation. However, it is important to note that high-proline variety development should focus on inducible production during stress rather than blocking degradation, which can cause toxic intermediate buildup [268]. On the other hand, knowledge on potential strategies to lower cellular osmotic potential to preserve turgor pressure is also applicable in breeding programs. This can be achieved by modulating soluble sugars (sucrose, fructose, and sugar alcohols) and glycine betaine (up to 157 µg g^−1^ FW in tolerant genotypes) contents [185]. In case of other stresses, like salinity, researchers have reported genotype-specific responses: tolerant varieties accumulate more proline than sensitive ones, while phenolic compounds e.g., flavonoid, contents increase, aiding metal chelation and oxidative stress mitigation. Therefore, future varieties must only be selected for higher osmolyte production but also for increased secondary metabolite production. Though, maintaining low Na^+^/K^+^ ratio remain a fundamental trait but alternative approaches such as higher reliance on osmolytes rather than higher Na^+^/K^+^ ratio in sensitive varieties should be used to protect cellular function [269]. In case of high temperature, the rapid induction of heat shock proteins (HSP70, HSP90) can be used as an early-warning system [270]. HSP alleles that can lead to faster and stronger expression can be prioritized in breeding programs. Similarly, it is necessary to look for genetic variation that stabilizes or prolongs the activity of key antioxidants like SOD and APX which decline sharply above 40 °C [38]. In breeding for prolonged heat stress, secondary metabolites like isoflavones (genistein, daidzein) that increasingly accumulate under prolonged heat stress, have proven to be a viable strategy that confers both antioxidant protection and membrane fluidity [271]. Other than yield, the qualitative changes as a result of heat stress such as inverse changes in lipid and protein contents is a challenge for breeding soybeans with optimum quality. This relationship can possibly be decoupled by targeting the key genes related to lipid biosynthesis (*GmBCCP2*, *GmKAS1*) and storage protein genes (*GmGy1*, *GmGy2*), and their regulatory networks [272]. The polyamine biosynthesis pathway is another prominent target for developing markers for selecting heat tolerant genotypes. To this regard, earlier research has revealed that spermidine levels can increase by 150% in heat-tolerant genotypes, which in turn stabilizes nucleic acids and membranes [272]. Contrasting to drought or heat, soybean plants experiencing waterlogging exhibit surge in ROS and NO in roots and leaves, with NO persisting in roots and both remaining elevated in leaves throughout waterlogging. Understanding the dual role of NO and ROS, both as signals and potential source of damage, is important for breeder’s understanding to select for a balanced response. Studies have shown that oxidative damage still occurs despite increased SOD and CAT activities, indicating inefficiency of antioxidant enzymes. Therefore, additional protective mechanisms should be explored to avoid photosynthetic apparatus damage and faster recovery of pigments. Fermentative pathways activate within 6 h, elevating lactate and ethanol. Nitrate reductase activity drives NO production in roots, while leaves utilize additional biosynthetic pathways [273]. The rapid activation of fermentation is a critical adaptive response. Selecting for genotypes that initiate these pathways more quickly or sustain them longer could provide the energy necessary for survival during prolonged oxygen deprivation. Reoxygenation transiently spikes ROS/NO in roots but normalizes leaf antioxidant enzyme activities and photosynthetic pigment levels within 48 h [273,274]. The recovery phase is as critical as the stress itself. Breeding targets should include not only the ability to survive waterlogging but also the capacity for rapid recovery upon re-oxygenation, as indicated by the swift normalization of antioxidant systems and photosynthetic pigments in leaves.

It is now clear from the above discussed literature that the antioxidant defense system is one of the key targets for soybean breeding for abiotic stress tolerance. This system’s effectiveness lies in its compartmentalized mechanisms, which must be considered in tandem. SOD isoforms convert O_2_^•−^ to H_2_O_2_, detoxified by CAT in peroxisomes and APX in chloroplasts. Researchers have reported a contrasting response to waterlogging with shoots showing 80% higher SOD activity but 67% lower root APX, exacerbating root oxidative stress [260]. This highlights that breeding efforts must aim for a balanced, organ-specific increased expression of these enzymes. Glutathione (GSH) pools deplete differentially—shoots lose relatively lower GSH than in roots. The maintenance of redox balance through the ascorbate-glutathione cycle, exemplified by 3:1 AsA:GSH ratios in tolerant genotypes, provides a precise biochemical benchmark for selecting resilient lines. Hormonal regulation integrates these responses. For example, the role of ABA at ≥0.5 µM upregulating proline biosynthesis genes (*P5CS*, *P5CR*) while suppressing *ProDH* [275] offers a clear hormonal pathway to modulate through breeding or management. Similarly, ethylene precursors such as ACC can be used as early indicators, as their concentration increase in waterlogged roots, correlating with aerenchyma formation and alanine aminotransferase activation. Moreover, Cross-talk between JA and SA fine-tunes antioxidants, with SA pretreatment boosting CAT activity under salinity [276]. Other biochemical adaptions reveal further precision targets, including alteration is stress-specific protein-levels. For example, drought stress induces higher histone H3 lysine 9 acetylation levels, which upregulate Delta1-pyrroline-5-carboxylate synthetase 1 in tolerant lines enhance proline synthesis [277]. This epigenetic modification points to a higher regulatory level that could be exploited to unlock downstream stress responses. Salinity stress causes the upregulation of *SOS1 Na^+^/H^+^* antiporters and *H^+^-ATPases* [278], providing direct gene targets for improving ion exclusion, while waterlogging increases the anaerobic polypeptides e.g., alcohol dehydrogenase, which peak at 12 h, shifting metabolism toward alanine [279]. Recent studies have reported the detailed understanding of the genetic stress regulators in soybean under stress scenario. For example, *GmDREB1* activates >15 osmoprotectant genes, and *GmNAC11* binds *SOD2/APX1* promoters under stress [109]. These master regulators are ideal candidates for genetic modification or marker-assisted selection to coordinately improve multiple downstream tolerance mechanisms. Similarly, several alleles from the wild soybean have been identified that enhance flooding tolerance via aerenchyma formation (PI 408105A) and adventitious root development [280], demonstrating the value of tapping into the wild gene pool to reintroduce beneficial, complex traits lost during domestication.

Metabolomic reprogramming is a major part of soybean’s biochemical response to abiotic stresses. In order to develop climate-smart soybeans, it is essential to understand the significant metabolic reprogramming that soybean plant initiates under stress maintain cellular homeostasis and ensure survival. Recent research has helped identify key biomarkers and pathways from stress tolerant/susceptible varieties. During hypoxia stress, energy metabolism shifts decisively e.g., pyruvate generated from glycolysis is diverted away from the TCA cycle and toward fermentative pathways, leading to increased activity of lactate dehydrogenase and alcohol dehydrogenase by 2.1- to 3.4-fold in roots and nodules, accompanied by elevated lactate and ethanol accumulation [274]. The magnitude of this shift makes these fermentation enzymes direct markers for selecting genotypes with superior energy maintenance. Similarly, enhancing the GABA shunt, which is activated under drought stress, can provide dual osmotic and metabolic benefits as it helps regulating the cytosolic pH and serves as a nitrogen storage intermediate [281]. Increased GABA and alanine in waterlogged roots sustain redox balance under hypoxic conditions, suggesting them as a broad-spectrum tolerance marker. Whereas, in case of heat stress, the disruption of the TCA cycle and the upregulation of the pentose phosphate pathway suggests the latter as an alternative route to bolster reducing power [282]. Drought-tolerant genotypes exhibit distinct metabolic adjustments, including changes in raffinose, which stabilizes membranes through hydrogen bonding (https://www.isaaa.org/kc/cropbiotechupdate/ged/article/default.asp?ID=19979; accessed on 13 April 2026), and increased accumulation of proline in leaves, functioning as both an osmolyte and a ROS scavenger. Among other key abiotic stresses, several marker metabolites like polyamines (spermidine and putrescine) in response to salinity stress [233], and γ- and δ-tocopherols in heat stressed soybeans provide discrete, measurable targets for breeding stress-resilient soybean varieties [283]. The list of metabolites that can be used as markers for selection is increasing. For example, in addition to flavonoid, hydroxycinnamates have been reported to scavenge ROS and reinforce cell walls [284]. Since, high temperatures (38 °C) suppress isoflavone biosynthesis by 46–86% in seeds due to downregulation of CHS7 and IFS2 genes [285], focus is also shifting towards other metabolic pathways. For example, abiotic stresses e.g., under heat stress, tolerant genotypes reduce 18:3 fatty acid content, thereby decreasing membrane fluidity [286]. Similarly, drought induces a 1.8-fold decline in phospholipids such as phosphatidylcholine, while free fatty acids like palmitate increase by 1.4-fold, indicating membrane degradation for energy salvage [284]. These metabolic shifts correlate strongly with stress tolerance outcomes, with resilient genotypes maintaining higher TCA cycle flux (citrate) and efficient amino acid recycling (e.g., glutamine) under salinity [282]. In contrast, sensitive genotypes exhibit prolonged sucrose accumulation but impaired glycolysis under waterlogging, ultimately leading to root necrosis [89]. The integration of these metabolic adaptations highlights the complex interplay between primary and secondary metabolism in soybean’s response to abiotic stress, providing potential targets for breeding stress-resilient varieties.

In short, soybean employs integrated morphological, physiological, biochemical and metabolic adaptations to combat abiotic stresses. Key strategies include root architecture modifications for water/nutrient access, osmotic regulation through proline/raffinose accumulation, antioxidant systems for ROS scavenging, and metabolic shifts like GABA shunt activation. These multilayered responses—mediated by stress sensors, hormonal signaling and genetic regulators—enable soybean to maintain productivity under drought, heat, salinity and waterlogging. Understanding these mechanisms provides critical targets for developing climate-resilient varieties through breeding and biotechnology approaches.

### 4.3. Increased Understanding of Biotic Stress Tolerance and Pathogen Dynamics Under Climate-Change Scenario

Improving soybeans resilience to new and re-emerging diseases is highly essential in order to achieve sustainable production in future. Current reliance on identifying pathogen-resistance genes provides a short-term protection in monoculture cropping system. Soybean breeders across the globe are experiencing difficulties in overcoming the re-emerging and new diseases under climate change scenario. In this regard, comprehensive atlas of more than 800 genes, alleles, and QTLs that confer resistance to 28 soybean diseases has been summarized by Lin et al. [287], providing a relatively up-to-date molecular-genetic framework for general and pathogen-specific resistance. This defense system operates through a sophisticated two-layered recognition mechanism. At the cell surface, pattern recognition receptors (PRRs) such as *GmDR1* detect pathogen-associated molecular patterns [288]. Intracellularly, a diverse array of nucleotide-binding domain leucine-rich-repeat receptors (NLRs; e.g., Rps11) mediates effector-triggered immunity by identifying pathogen virulence factors [289]. Recent advances in protein engineering and structural biology have revealed that certain receptors can recognize a broader spectrum of pathogens. For instance, the *RXEG1* receptor-like protein confers broad-spectrum resistance in both soybean and cotton by specifically targeting microbial glycoside hydrolase 12 proteins without compromising yield [290]. These findings highlight the potential of receptor stacking strategies combining NLRs and PRRs to create more durable resistance. However, engineering such multi-layered defenses requires precise regulation of immune receptors to avoid unintended autoimmunity responses that could lead to necrosis and reduced plant vigor. Emerging knowledge of receptor interaction networks and signaling architectures may also enable novel strategies to reduce post-harvest seed spoilage by pathogens. For detailed discussions of soybean immunity mechanisms, readers are directed to comprehensive reviews by Lin, Chhapekar, Vieira, Da Silva, Rojas, Lee, Liu, Pardo, Lee and Dong [287] and Rao, et al. [291].

For future soybean breeding targeting enhanced biotic stress tolerance, the must have resource is to have access to diverse genetic variation. Particularly from related species e.g., wild soybean, or those affected by same pathogens. These undomesticated gene pools harbor alleles lost during breeding and are critical for introducing novel resistance [292]. A range of genetic resources have been successfully identified by employing omics studies e.g., genome assembly, re-sequencing the soybean populations and wild soybeans, transcriptome sequencing, and epigenetic sequencing [293,294]. Beyond accessing genetic resources, sustainable soybean production under climate change requires understanding pathogen population dynamics and host responses to interacting stresses (https://news.siu.edu/2005/11/110105kj5127.php; last accessed on 19 June 2025). Trade-offs exist; for example, breeding for drought tolerance may inadvertently affect susceptibility to SDS. Additionally, eCO_2_ has pathogen-specific effects—enhancing resistance to *P. syringae* pv. *glycinea* but increasing vulnerability to viral pathogens [59]. Breeding programs should therefore prioritize viral resistance under future atmospheric conditions.

Climate models predict significant shifts in pathogen biogeography, with warming temperatures expanding the range of soybean cyst nematode into northern latitudes such as Quebec, Canada [95]. Warmer winters may also enable the survival of traditionally southern pathogens like the charcoal rot and frogeye leaf spot agents in new regions. Rising soil temperatures could accelerate nematode reproduction rates, while increased precipitation may elevate pressure from fungal pathogens and insect vectors of viral diseases. These predictions allow breeders to anticipate future threats and preemptively incorporate resistance to historically “southern” or “tropical” pathogens into materials destined for higher latitudes. Although some pathogens like rust may decline in severity under projected climates [59], the net effect of these changes will likely be an overall increase in disease pressure, compounded by potential interactions with other stressors such as drought and heat. Critically, the impacts of climate change on the efficacy of both genetic resistance and chemical control measures remain poorly understood [295]. This knowledge gap is a priority for research; without understanding how elevated temperatures, CO_2_, and drought alter gene expression and fungicide performance, breeding and management strategies risk obsolescence. Adaptive management, combining genetic diversity, predictive modeling, and flexible control tactics, will be essential for future soybean production systems.

### 4.4. Optimizing Water Use Efficiency and Sustainability in Soybean Production

Based on the comprehensive research progress documented in the literature, there are seven key actionable strategies for optimizing WUE and sustainability in soybean. Soybeans, as C3 plant, inherently exhibit higher transpirational water loss, making WUE a key target for breeding programs. Generally, efficient water use can be achieved through three primary strategies: increasing yield without additional water input, reducing water use while maintaining yield, or a combination of both. In regions like the North China Plain, where water scarcity is exacerbated by intensive irrigation, shifting from water-intensive cereals to legumes like soybeans can improve system-level WUE. The first strategy through which breeders can improve soybean’s WUE is through genetic improvement, though soybean’s higher water consumption per leaf area poses challenges. Genetic improvements in WUE are essential to mitigate these limitations, with carbon isotope discrimination (Δ^13^C) and C isotope composition (δ^13^C) serving as a reliable proxy (markers) for WUE due to their high heritability and strong genetic control. The already identified QTLs associated with Δ^13^C and other drought-related traits, such as canopy wilting and root architecture, can be prioritized through marker-assisted selection for improved WUE [296,297]. Similarly, breeders may target genotypes with conserved water use phenotypes, characterized by lower root length density and restricted transpiration, as these traits consistently confer superior yield stability and WUE under terminal drought, as demonstrated in cultivars like J19 and ZH [298]. The second strategy involves irrigation management. Agronomists and farmers must adapt regulated deficit irrigation strategies targeting 70–80% field capacity, as this optimizes the trade-off between yield and WUE, with peak productivity observed at a crop water stress index of 0.34 [299]. While partial root drying can enhance WUE by up to 48% compared to conventional deficit irrigation, extreme water deficits (e.g., 40% replacement of field capacity) risk significant yield losses [298]. The third strategy involves soil management and amendments. In this regard, biochar application should be optimized—applied at rates that improve soil water retention and nutrient availability without inducing hydrophobicity that reduces WUE. Sustainability can be achieved by reducing input requirements and adapting to cropping systems. In Brazil and the USA, the top two soybean producers, average total direct cost per acre is 390 and 295 US $, respectively [300], whereas the additional costs on drying, storage, transportation, and crop insurance leads to further increase. In terms of cost reduction, strategies such as optimizing soil health, using high-yielding and pest-resistant varieties (e.g., by using soybean cyst nematode-resistant soybean varieties for six years resulted in a farmers’ welfare gain of 324.77 million US $ [301]), adjust planting practices, implement effective weed and pest management, and manage nutrients and water efficiently. Use of local alternative DAP inputs e.g., cow dung manure and ashes, helped Kenyan farmers to double the labor productivity [302]. Fourth strategy involves BNF and nutrient management, which further help in cost reduction (see Section 4.1 for strategies on improving BNF); it provides ecosystem services with high economic values by saving billions of US $ in terms of N-urea and avoiding multiple million Mg CO_2_-equivalent. For example, in Brazil, BNF saved 15.2 billion US $ and avoided 183 million Mg CO_2_-e [303]. Fifth strategy can be cropping system optimization. Farmers should implement precision seeding at rates (120,000 seeds/acre), as a decade of Nebraska research confirmed saving 10.69 US $/acre savings without affecting yield (https://cropwatch.unl.edu/2017/10-years-research-show-benefit-reducing-soybean-seeding-rates/; last accessed on 19 June 2025). To further enhance stability, crop rotation and relay intercropping, which have helped farmers to maintain soil fertility, increase nitrogen recovery efficiency, and reduce N input [304]. Complementing these practices with integrated pest management strategies can cut input cost by reducing insecticide application on soybean by 50% [305] (https://news.agropages.com/News/NewsDetail---48917.htm; last accessed on 19 June 2025). Finally, leveraging precision agricultural technologies guided by positive associations between conservation practices and long-term profitability [306] will help optimize inputs and maximize economic returns over time. Sixth strategy should focus on managing the emerging pollutants (Figure 4), where integrated remediation methods combining biochar, microbial inoculants, and targeted nutrient supplements can be used. Finally, the next strategies should focus on phenomics as discussed below.

### 4.5. Accelerating Breeding Through Phenomics

High-throughput phenotyping (HTP) is emerging as a transformative tool in soybean breeding, addressing the challenges posed by genetic diversity and genotype-by-environment (G × E) interactions while improving the precision and efficiency of selection strategies [307]. There are several ways through which breeders can leverage advances in HTP. (1) Breeders may prioritize the integration of unmanned aerial vehicles (UAVs) equipped with optical sensors, particularly RGB cameras, which have enabled large-scale, non-destructive assessment of canopy dynamics, biomass, and stress responses [308,309]. RGB-based vegetation indices (VIs), such as the Normalized Green-Red Vegetation Index and Green Leaf Index, should be routinely used to quantify photosynthetic efficiency, biomass, and water stress. Temporal VI analysis should be adapted to reveal growth patterns critical for understanding genotypic adaptability. (2) Early stress detection by the use of machine learning (ML) together with the use of multispectral and hyperspectral sensors [310]. Deep learning models, trained on publicly available datasets like PlantVillage (54,000+ images), should be adapted and validated for local germplasms [311]. (3) We suggest that breeders may invest in emerging below-ground phenotyping platforms for root characterization, X-ray computed tomography for high resolution root imaging, and transparent soil systems for controlled-environment RSA studies [310,312]. These tools should be deployed to elucidate the genetic basis of nutrient uptake efficiency, drought tolerance, and water use efficiency—traits intrinsically linked to root architecture. A critical gap exists in the assimilation of intermediate HTP-derived traits into crop models for yield prediction. (4) Researchers must prioritize the development of data assimilation techniques that couple vegetation indices and canopy traits with process-based models to refine yield forecasts under variable environments [313]. Explainable artificial intelligence methods should be employed to interpret spectral signatures and identify the most predictive traits for target environments, enhancing the transparency and adoption of HTP-based selection strategies. (5) Value added breeding requires integration of proximal sensing for seed composition traits, including protein and oil content, as well as end-use quality metrics such as estimated processed value [310]. Near-infrared spectroscopy and hyperspectral imaging should be calibrated and deployed on harvest equipment or in laboratory settings to enable high-throughput assessment of seed quality traits, facilitating simultaneous selection for yield and nutritional or industrial quality. (6) A key focus should be adaptation of multi-sensor integration, leveraging UAV-ground robot synergies for 3D canopy reconstruction and root phenotyping [310,312]. And finally, (7) to fully realize the HTP’s potential, collaborative phenomics networks must be established to address data standardization, scalability, and interoperability. This will require shared protocols for sensor calibration, data collection, and trait extraction across environments. Public-private partnerships should be fostered to develop and maintain centralized databases linking HTP-derived traits with genomic and phenotypic data [310].

### 4.6. New Technologies and Tools: Lab to Field

Significant developments in sequencing technologies have helped breeders to refine high-quality genome assemblies of *G. max* and *G. soja* [314,315]. Resequencing has provided detailed insights into the role of structural variations in soybean improvement [316] and identified domestication related genes [317]. A recent review presented a detailed literature survey of omics and functional genomics studies on soybean, and provided a decadal vision [318]. Key future directions to achieve the higher and sustainable soybean yields by 2050 and beyond, it is essential to harvest the benefits of modern technologies i.e., precision breeding, GS, biotechnological interventions, increasing phenotyping accuracy, pan-genomics, transcriptomics, proteomics, metabolomics, epiomics, synthetic biology, nanomaterials, etc. [319]. At the genomics front, it is essential to translate the data and information from lab to field. In this regard, whole genome sequencing combined with high-density-marker QTL mapping by resequencing has unveiled several genes e.g., *GmCHX1* for salt stress tolerance [320], *GmSW17* for seed size [116], Bloom1 for seed coat bloom and oil content in wild soybean [321], *Gm18GRSC3* conferring soybean mosaic virus resistance [322], etc. CRISPR-Cas can be leveraged to translate such genomic results to field e.g., editing of *GmARM* improved resistance to multiple stresses in soybean [323]. Such developments provide detailed functional insights and allow their integration in breeding programs tailored for sustainable production under climate change scenario. Such strategies have been reviewed and discussed in earlier reviews in relevance to salinity [278], plant architecture and yield [114,324], disease resistance [325], drought stress in BNF [326], and heat stress [272]. Such technologies are increasingly being aided by ML and artificial intelligence (AI) [327].

Past two decades have significantly increased the soybean omics data enabling detailed gene and transcript exploration and meta-analyses under stress scenarios [328]. Several databases focusing soybean research are providing a wealth of underexplored proteomic, genomic, expression, QTLs, and related data e.g., SoyBase (https://www.soybase.org), SoyOmics (https://ngdc.cncb.ac.cn/soyomics/index; accessed on 9 April 2026), SoybeanGDB (https://venyao.xyz/soybeangdb; accessed on 9 April 2026), Soybean Expression Atlas (https://soyatlas.venanciogroup.uenf.br/), Soybean proteome database (http://proteome.dc.affrc.go.jp/Soybean/; accessed on 9 April 2026), SoyKB (https://soykb.org/), etc. Sequencing technologies such as next generation sequencing, long-read sequencing, Iso-seq, etc., and increasingly being adapted to develop methods such as SLAF-seq [329], RAD-seq [330], GBS [331]. These methods have enabled rapid identification of resistance genes, genomic prediction, and QTLs associated with agronomic traits e.g., flowering time and seed size. Large-scale QTL mapping and GWAS studies are adapting genome-scale sequencing, however, progress is need to resolve the imperfections such as large candidate regions [139,319]. Moreover, the fact that a large portion of soybean genome comprises of non-coding region, possibly containing crucial regulatory elements for gene expression, soyENCODE project can take advantage of multiomics datasets to characterize the regulatory elements [318]. Additionally, the availability of soybean and wild soybean pan-genomes is expanding evolutionary and functional genomics research [332]. This avenue is being further expanded to sup-pangenome level enabling understanding the perenniality-to-annualilty transition [333]. Nevertheless, the information about the stress tolerance genes/QTLs/alleles from the perennials and the structural variations in (super)pan-genome can be integrated in ongoing soybean breeding programs for improved resilience to stresses. Developments in sequencing technologies have also advanced transcriptional and post-transcriptional regulation of genes. A quick search (as of 20 June 2025) in sequence read archive of NCBI by using keywords “soybean” and “*Glycine max*” revealed 36,521 BioSamples, 1473 BioProjects, and 4698 GEO datasets. This data, when integrated with computational approaches, is being used to construct gene expression networks to infer gene functions, evolutionary analyses, and functional relevance [334] and references therein. While most BioProjects targeted spatio-temporal transcriptome studies involving organs and tissues and particular developmental stage or stress scenario, single-cell omics is enabling the identification of distinct cell types and a large number of accessible chromatin regions [335]. Single-cell transcriptome atlases e.g., of soybean roots and mature nodules, have lead the identification of novel nodulation related genes e.g., *GmFWL3* [336]. The large-scale single-cell omics datasets (atlas) have been utilized for the development of user-friendly databases [337]. However, parallel advancements in computational tools, lack of standard guidelines on the biological replicates, treatment, processing, data acquisition, and formal analysis are existing challenges. Additionally, integration of this large-scale data in ongoing and future breeding program and ultimate translation to field remains an area of further exploration. Transcriptome knowledge can further be supplemented by proteome and metabolome analyses. While soybean metabolomics is well established in terms of understanding the sensing and adaptive responses under stresses [338], the proteome is least explored. Proteomics offers advantages over other omics approaches such as knowledge on post-translational modification. As of now, UniProt (Proteome ID UP000008827) hosts the reference proteome derived from the *G. max* genome published in 2010 [339]. With the availability of updated and near complete genome assemblies [316], reference proteome can be updated to maintain an accurate and comprehensive representation. Proteomics has been successfully applied for the identification of signaling, amino acid biosynthesis, oxidative phosphorylation, glycolysis, energy metabolism, cell wall, and many other processes related regulatory protein expression under flooding, salt, and phosphorus treatment [340,341,342]. These and other proteome studies have allowed the identification of stress and/or growth-related marker proteins. While, these omics techniques provide sufficient resolution of growth and development or stress scenario, a complete picture of the perception, signaling, responses, and adaption of soybean can be capture by integrating multi-omics techniques [319]. For example, an integrated transcriptomics, proteomic, and metabolomic analysis provided detailed understanding of the molecular mechanism of salt stress tolerance in soybean [343]. Future studies should focus on the integration of multi-omics under complex/combined stress scenarios and translate the results at field level. To this regard, systems biology, AI (ML) frameworks can help soybean breeders for feature engineering for stress tolerance gene discovery from multi-omics data [344]. It is also important to note that, the major omics have ascended to multi-epiomics (epigenomics, epitranscriptomics, and epiproteomics), enabling detailed understanding of complex regulatory networks governing functions related to plant growth, development, and stress tolerance [345]. Among these epigenomics has emerged as the major sub-field and has enabled the identification dynamic epigenetic differences in soybean in response to low-phosphorus [346], growth and development and stress tolerance [347]. Whereas, epitranscriptomics is also being largely adapted, for example, to explore the role N6-methyladenosine (m6A) modification of messenger RNA (mRNA) in light responses [348], rhizobia driven root growth under cadmium stress [349], and other abiotic stress responses [350]. Since the stress responses are complex, future studies may integrate mRNA modification with ncRNA networks and hormone signaling and biosynthetic pathways.

In Section 4.5 we discussed how HTP can be a tool to accelerate breeding. Additionally, it is important highlight that the translation of the results of the integrative multi-omics studies can fully be utilized by leveraging the use of phenomics. Particularly the HTP of morphological, biochemical, and physiological traits in a non-destructive and non-invasive way. Furthermore, the AI, ML, combined with statistical genomic methods in smart-farms are an area of extreme interest. These technologies generate big data, for which selecting appropriate models/methods for data processing, integration, and analyses is an underexplored. To improve the efficiency of phenome-phenome association studies, the use of hyperspectral wide association studies, in the combination with conventional GWAS and ML have proven to be advantageous in selection of superior genotypes for yield [351] and salt tolerance [352]. Thus, in future HTP, AI (ML), and integrative multi-omics studies are likely to increase soybean yield predictability under a climate-change scenario.

Biotechnological interventions, including CRISPR/Cas, synthetic biology, RNA interference, gene transformations have proven to be most used tools for engineering adaptation and tolerance in soybean. As of 2024, the global cultivated area of GM soybean reached 105.1 million hectares (https://gm.agbioinvestor.com/; last accessed on 21 June 2025), indicating transformative role of transgenic soybean breeding during the last decade [353]. CRISPR/Cas9 is being rapidly adapted in soybean molecular breeding read [354] for more details. However, considering restrictions on GM crops in many countries, techniques such as DNA-free and genotype-independent CRISPR/Cas9 system have been developed to bypass the regulatory restrictions [355]. Adaption of this technologies can speed up the variety approval and commercialization process. Future holds the potential use of synthetic biology such as the usage of expression elements, ribosomes binding sites, protein degradation tags for optimizing the gene expression. Interventions such as protein engineering, synthetic scaffolds, modular engineering, and genetic circuits can be leverage to optimize metabolic pathways, improve stress tolerance, protein and oil contents [356]. When combined with speed breeding, the biotechnological tools, may offer an accelerated soybean variety development program. To this regard, soybean significant progress has been made where four to five soybean generations per year can be achieved by manipulating the photoperiod and light regimes [357,358].

Overall, developments in omics technologies, gene editing tools, and AI-driven approaches have significantly accelerated soybean improvement, enabling precise trait manipulation and faster breeding cycles. While current efforts have successfully identified key genes for stress tolerance and yield components, future work must focus on translating these discoveries into field applications through integrated multi-omics pipelines and predictive modeling. Critical to this translation will be enhancing selection accuracy through improved phenotyping methods, strategic stacking of major and minor genes, and optimizing selection intensity. The development of non-GMO genome editing systems and climate-resilient smart varieties will be crucial for global adoption, requiring careful management of genetic variance and reduced recycling time in breeding programs. As soybean research advances, combining pan-genome resources with high-throughput phenotyping and ML will be essential to overcome yield plateaus and meet 2050 demands. However, realizing this potential depends on overcoming persistent challenges in data integration, regulatory harmonization, and field validation to ensure these technological breakthroughs translate to sustainable production gains.

## 5. The Future Soybean Breeder: A Hybrid Orchestrator of Data and Dirt

**Figure 6:**
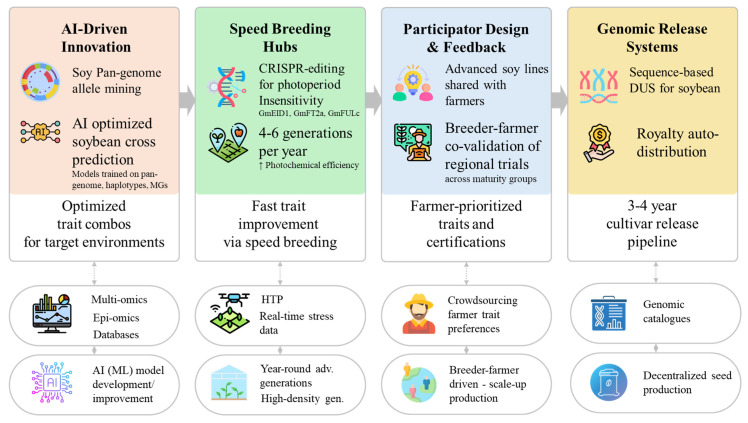
Future of soybean cultivar delivery framework integrating AI-driven innovation, speed breeding, participatory design, and genomic release systems. Key components include (i) AI-assisted pan-genome mining and cross optimization, (ii) speed breeding hubs enabling 4–5 generations per year using CRISPR-edited photoperiod insensitivity, (iii) farmer participatory design for trait prioritization and co-validation and (iv) genomic release systems using sequence-based DUS replacement and blockchain-tracked royalty distribution.

It is important to highlight the key tools and skills that a breeder would need to breed soybean varieties for future climate-change scenarios. In 2003, Jonathan Knight highlighted that the classical field breeding was being supplanted by high-tech methods [359]. Has this been manifested in today’s soybean research? And what will be the path to 2050 demands? To our understanding a synthesis of both classical and modern high-tech paradigms hods the answer. As Bassi, et al. [360] noted, future breeding will likely bifurcate: routine tasks (e.g., genomic preselection, pedigree tracking) will possibly become fully automated through AI and service-based platforms, while the breeder’s role shifts toward strategic trait deployment and participatory design with farmers/industry. For soybean, this may mean CRISPR-edited stress resilience genes are used algorithmically, but field validation remains a challenge for complex traits like stress adaptations or soil-microbe interactions—aligning with Repinski, et al. [361] call to “revive the art” of observation. By 2050, soybean breeding programs may operate as closed-loop systems where multi-omics data and farmer feedback continuously refine gene-edited pipelines, yet Knight’s fear of “lost field wisdom” could reemerge if institutional knowledge isn’t preserved and updated. The challenge lies in balancing “service-based breeding” with the socio-agronomic complexity that soybean cultivation demands—ensuring technology serves sustainability without disconnecting breeders from the fields that birthed the discipline. These skills will enable soybean breeders to deliver cultivars for future, as proposed by Bassi, Sanchez-Garcia and Ortiz [360], in a framework based on participatory approaches (Figure 6).

To realize this vision, the soybean breeders of future must become a multidisciplinary specialist, equally equipped with advanced technologies and empirical field knowledge. Core technical skills will include mastery of precision breeding tools (GS, haplotype-based breeding), CRISPR-Cas systems for editing climate resilience traits, and pan-genome utilization to unlock alleles from wild relatives, particularly *G. soja* [292,362]. They’ll need expertise in high-throughput phenotyping (drones, hyperspectral imaging) and AI-driven predictive modeling (Python version 3.14.4, R version 4.5.0) to analyze multi-omics data, while maintaining hands-on knowledge of stress physiology and soil-microbe interactions that algorithms might overlook. Beyond technical/methodological expertise, success will require systems thinking: designing agriculture traits (improved nutrient uptake, rhizobium symbiosis), navigating global gene-editing regulations, and collaborating across disciplines—from climate modelers to socio-economists—to align varieties with market needs and regenerative practices. While automation handles routine selection, breeders will focus on strategic pipeline design, optimizing resource allocation through engineer-like precision while maintaining the skills for field observation. Critical upskilling priorities include mastering CRISPR workflows (sgRNA design to off-target validation), contributing to open-data initiatives (SoyENCODE), and engaging farmers in participatory breeding to ensure practical relevance. This hybrid role—part data scientist, part agronomist, part strategist—will be crucial for developing climate-smart varieties that meet 2050’s dual demands of sustainability and productivity, all while keeping the breeder’s irreplaceable field wisdom at the heart of innovation.

## 6. Conclusions and Future Recommendations

The challenges facing global soybean production represent one of the most complex scenarios in agricultural adaptation. Our comprehensive literature survey, synthesizing over 400 sources, has identified multiple interconnected constraints. Yield stagnation exists across 23% of the global harvest area, including major production regions such as China, India, and the US Corn Belt, Paraguay, Brazil, Argentina, and Russia, with widening yield gaps across 35% of the production zones. Average farm yields remain 2770–2790 kg ha^−1^ globally, leaving 44% of the production potential unrealized. Climate change exacerbates these challenges through multiple pathways: temperature extremes cause 45.5% yield loss above 28 °C; combined with 28–74% losses from drought, 17–56% from flooding, and salinity reduced filled pods by 55–65%. Combined stresses, particularly heat and drought, inflict synergistic losses of 64–91%, far exceeding the individual stresses. Biotic stress is intensifying, with projected yield losses of 11–32% from pests and diseases under climate change, driven by poleward pathogen migration, altered host physiology, and pathogen evolution. The CO_2_ paradox adds further complexity as it boosts photosynthesis but reduced seed protein content, alters oil composition, and fails to compensate for extreme climate change impacts. We conclude that global food security and economic stability are in danger due to the combination of yield stagnation, growing climate challenges, and systemic production hazards. The urgent need for innovative methods that incorporate these existing technologies into cohesive breeding and production systems is highlighted by the fact that current average yields only represent 19.4% of theoretical potential.

Looking ahead, several critical pathways emerge for developing climate-resilient soybean systems. First and foremost, we must leverage the power of modern breeding technologies while maintaining connection to field realities. The integration of pan-genomics with advanced phenotyping offers unprecedented opportunities to mine the untapped genetic diversity of wild soybean relatives, particularly for complex stress tolerance traits. CRISPR-based gene editing, especially when combined with AI-driven prediction models, enables precise manipulation of key pathways—from thermostable flowering (*GmELF3*) to salt exclusion mechanisms (*GmCHX1*). However, as the mixed success of controlled-environment studies versus field performance demonstrates, these technologies must be validated under real-world conditions through extensive multi-location trials. The emerging field of single-cell omics presents particular promise for understanding tissue-specific stress responses that could unlock new breeding targets. Furthermore, to capture complex field interactions, future research must prioritize the integration of multi-omics platforms (transcriptomics, proteomics, and metabolomics) under combined stress settings. The identification of stress tolerance genes from these datasets will depend on systems biology and AI-driven frameworks. Simultaneously, combinatorial techniques should replace single-agent applications in mitigation strategies. The most adaptable approach is microbial inoculation with PGPR, rhizobia, and endophytes, especially when paired with nutrient supplementation (silicon, selenium, calcium), nanoparticles, or charcoal to create synergistic stress tolerance. It is a top research priority to validate these combinatorial treatments in various field settings.

Equally important is the need to develop integrated stress management strategies that account for the complex interactions between abiotic and biotic factors. Our review highlights how climate change alters pathogen dynamics, with diseases like SDS thriving under warmer, wetter conditions while charcoal rot dominates in drought scenarios. Future breeding must therefore move beyond single-stress solutions to develop varieties with combined tolerance packages. This requires innovative screening protocols that simulate climate change scenarios—for instance, testing soybean responses to eCO_2_ combined with heat waves or alternating drought-flood cycles. The development of “climate analogue” testing sites that mirror predicted 2050 conditions in different regions could provide invaluable data for trait selection.

The soybean-rhizobia interaction represents another frontier for soybean improvement. Emerging evidence suggests that rhizobia and soybean engineering and targeted consortia application could enhance stress resilience while reducing input dependency. Future research should prioritize understanding how to optimize these interactions under climate stress, particularly for nitrogen fixation efficiency in marginal soils. The combination of elite soybean cultivars with stress-adapted rhizobium strains could provide a sustainable pathway for maintaining productivity in challenging environments.

On the agronomic front, precision adaptation strategies will be crucial for closing yield gaps. Our review shows that simple interventions like optimized planting dates could recover 18% of yields in tropical regions, while advanced water management techniques may mitigate drought impacts. However, these solutions must be tailored to local contexts through participatory research with farmers. The development of decision-support tools that integrate real-time weather data, soil conditions and variety-specific recommendations could dramatically improve adoption rates.

The institutional and policy dimensions require equal attention. Current variety release systems, often requiring 10+ years for testing and seed multiplication, are inadequate for the speed of climate change. We propose a transition to genomic-based release systems where varieties are characterized by their haplotype fingerprints and performance predictions, enabling faster deployment of climate-adapted material. Simultaneously, international cooperation must be strengthened to ensure equitable access to breeding technologies, particularly for public sector programs in developing countries.

For the breeding community, several specific actions should be prioritized:

- Establishment of international pre-breeding consortia to systematically introgress wild soybean alleles into elite backgrounds.
- Development of open-access phenotyping platforms for climate stress screening.
- Creation of a global soybean innovation fund to support public-good breeding research.
- Implementation of farmer participatory networks for trait prioritization and variety testing.
- Advancement of regulatory science to enable responsible use of gene editing in public breeding programs.

The human dimension remains paramount. As technology advances, we must guard against the “lost field wisdom” that Jonathan Knight warned about two decades ago. The soybean breeders of 2050 will need to be multi-talented—equally conversant in computational biology and field pathology, in gene editing and extension agronomy. Training programs must evolve to create this new generation of “complete breeders” who can integrate across disciplines.

Ultimately, the success of these efforts will be measured not just in yield metrics but in real-world impact. Can we develop soybean systems that maintain productivity while using fewer resources? Can we create varieties that help smallholder farmers weather climatic worsen? Can we build equitable innovation systems that share benefits broadly? The answers to these questions will determine whether soybean remains a pillar of global food security or becomes another casualty of climate change.

The window for action is narrow but remains open. With coordinated effort across sectors and disciplines, we can rise to meet the existing and forthcoming challenges in soybean production. The strategies outlined here provide a roadmap, but their implementation requires urgent commitment from researchers, policymakers, industry leaders and farming communities worldwide. The time for incremental improvement has passed—what’s needed now is nothing short of a revolution in how we breed, grow and share this vital crop.

## Figures and Tables

**Table 1:** Estimated genetic gain for soybean yield in major producer countries.

Country	Approximate Genetic Gain Rate	Resources
Brazil	12–46 kg ha^−1^ yr^−1^ Up to 84 kg ha^−1^ yr^−1^ (specific regional studies) ~39.4 kg ha^−1^ yr^−1^ (over 1960–2021)	[13,15,16,17]
United States of America	18–40 kg ha^−1^ yr^−1^ (1989–2019) ~8.7 kg ha^−1^ yr^−1^ (1923–2008)	[18,19]
Argentina	20.5–46.1 kg ha^−1^ yr^−1^ (last 15 years) ~32.2 kg ha^−1^ yr^−1^ (over 1960–2021)	[15]
China	~17.4 kg ha^−1^ yr^−1^ (Henan province) ~5.8–16.2 kg ha^−1^ yr^−1^ (Northeast China, 1923–2008)	[12,20]
India	~22–23 kg ha^−1^ yr^−1^ (1969–2008)	[21,22]
Canada	~10–11 kg ha^−1^ yr^−1^ between 1934 and 1992 Up to 30 kg ha^−1^ yr^−1^ between 1976 and 1992 Up to 26 kg ha^−1^ yr^−1^ were reported in uniform tests up to 2001 ~1% annually (Southern Ontario)	[23,24]
Paraguay	Consistent yield increases noted as more land is allocated to the crop	[25,26]
Bolivia, Russia, Ukraine, Uruguay	Specific, quantifiable genetic gain rates are not easily found in search results. Russian sources mention variety development and a positive trend in production but lack quantitative genetic gain data.	Russia [27,28] Ukraine [29,30] Bolivia [31] Uruguay [32,33]

## Data Availability

All studied reviewed have been cited. All databases accessed for information have been cited or their links and access dates are provided.
